# Lymph-Node Inspired Hydrogels Enhance CAR Expression and Proliferation of CAR T Cells

**DOI:** 10.1021/acsami.4c19942

**Published:** 2025-03-05

**Authors:** Miquel Castellote-Borrell, Marc Domingo, Francesca Merlina, Huixia Lu, Salut Colell, Mireia Bachiller, Manel Juan, Sonia Guedan, Jordi Faraudo, Judith Guasch

**Affiliations:** † Dynamic Biomaterials for Cancer Immunotherapy, Max Planck Partner Group, Institut de Ciència de Materials de Barcelona (ICMAB-CSIC), Campus UAB, Bellaterra 08193, Spain; ‡ Soft Matter Theory Group, Institut de Ciència de Materials de Barcelona (ICMAB-CSIC), Campus UAB, Bellaterra 08193, Spain; § Department of Physics, Universitat Politècnica de Catalunya-Barcelona Tech (UPC), Barcelona 08034, Spain; ∥ Department of Hematology, Hospital Clinic, Institut d’Investigacions Biomèdiques August Pi i Sunyer (IDIBAPS), Barcelona 08036, Spain

**Keywords:** biohybrid hydrogels, CAR T cells, CAR expression, proliferation, molecular dynamics

## Abstract

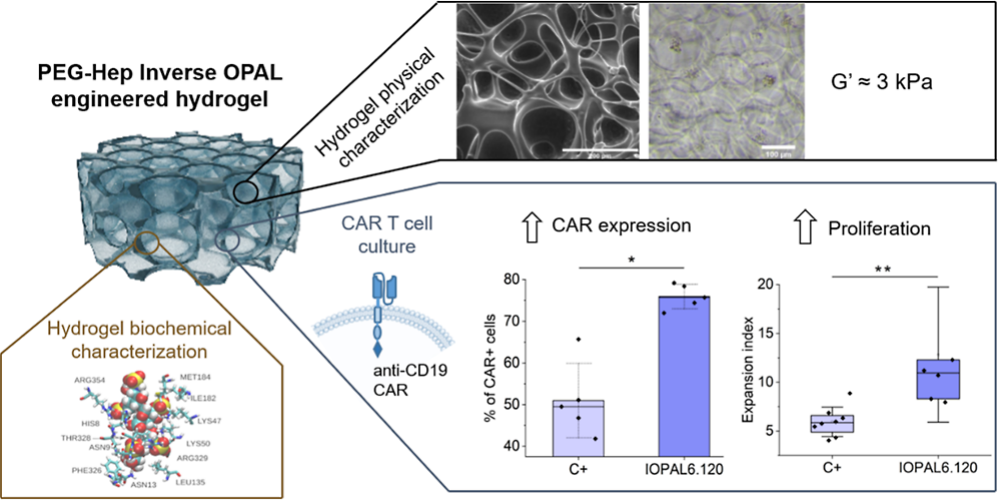

Chimeric antigen receptor (CAR) T therapy has shown unprecedented results in clinical practice, including long-term complete responses. One of the current challenges of CAR T therapy is to optimize its production in order to lower its cost. Currently, the in vivo activation of T cells by dendritic cells is replicated ex vivo using polymeric magnetic beads coated with antibodies to induce polyclonal T cell activation. However, current practice overlooks the importance of the complex environment that constitutes the lymph nodes, in which T cells activate and proliferate in vivo. Hydrogels are an ideal candidate material for mimicking the properties of natural tissues such as lymph nodes. In this study, key conditions of the composition, stiffness, and microarchitecture of hydrogels were experimentally and theoretically investigated to optimize primary human CAR T cell culture, focusing on CAR expression and proliferation. Poly(ethylene glycol)–heparin hydrogels featuring interconnected pores of 120 μm and an intermediate stiffness of 3.1 kPa were identified as the most suitable conditions for promoting CAR T cell expression and expansion. Specifically, these hydrogels increased the percentage of CAR+ cells by 50% and doubled the replication index compared to suspension cultures. In conclusion, these newly engineered hydrogels are an interesting tool to help improve CAR T cell manufacture and ultimately advance toward a broader clinical implementation of CAR T cell therapy.

## 1 Introduction

Adoptive cell therapy (ACT) is a novel immunotherapy that is achieving unprecedented results in oncology, including long-term remissions of relapsed and refractory cancers.^1−3^ It consists of harnessing the power of the immune system by using immune cells, usually autologous T cells, as “living” drugs after ex vivo conditioning. In particular, T cells are harvested from the patients, genetically modified or selected, and then expanded in vitro. Once an adequate number of therapeutic cells is reached, they are reinfused into the patients to mediate cancer cell destruction. A challenge of ACT is to manufacture enough and adequate therapeutic T cells to obtain long-lasting clinical responses.^1,3^

Chimeric antigen receptor (CAR)-T therapy is an ACT approach that consists of genetically modifying T cells to express an artificial receptor, the CAR, that selectively targets cancer cells.^3,4^ Specifically, a viral vector is used for the transduction of T cells with the CAR of interest during their expansion in vitro. Anti-CD19 CAR T therapies against cancerous B cells occurring in leukemia and lymphoma have been approved by the FDA and EMA and are currently used in clinical practice with good results.^5,6^ Despite such success, some challenges lie ahead, such as the optimization of the costs related to these therapies. Currently, viral transduction and cell expansion steps are two of the highest costs involved in CAR T therapy. Therefore, their optimization is key for CAR T therapy to become a broadly used therapy.^7^

In vivo, resting T cells are activated in the lymph nodes upon their interaction with antigen presenting cells (APCs) through the immunological synapse (IS).^8,9^ Once activated, T cells start to proliferate, aided by the architectural and biological cues that the lymph nodes offer.^10−12^ However, ex vivo T cell expansion methods have so far only focused on mimicking the activation provided by APCs.^13,14^ Even nanoscale control of the spatial distribution of such signals has been explored to efficiently mimic the IS.^15−19^ Nevertheless, the most common approach and current gold standard for the ex vivo polyclonal activation of T cells is the use of polymeric magnetic beads coated with anti-CD3 and anti-CD28 antibodies (e.g., Dynabeads, Thermo Fisher Scientific).^20^ Dynabeads offer a potent T cell activation, but they alone fail to reproduce the complexity of the in vivo environment in which T cells activate and proliferate. In fact, such strong T cell activation has been shown to negatively affect cell viability.^21,22^

To tackle this deficiency, some efforts have been made to engineer materials that can mimic the properties of lymph nodes.^14,23,24^ Among them, we showed that hydrogels made of poly(ethylene glycol) cross-linked with heparin (PEG–Hep) could improve the expansion of primary human CD4+ T cells by providing adequate mechanical and biochemical stimuli to the cells.^23^ In a more advanced study, a second generation of PEG–Hep hydrogels were engineered with precise pore size and enlarged interconnectivity,^24^ through the inverse OPAL (IOPAL) technique. These IOPAL hydrogels showed improved viability and proliferation of primary human CD4+ T cells compared to unstructured or bulk hydrogels and suspension systems.

CAR T cell manufacturing involves a genetic modification of T cells, which is commonly performed with lentiviral vectors, i.e. lentiviruses (LV) used to genetically modify cells. For gene delivery purposes, different generations of LV have been designed to improve their safety and transduction efficiency.^25^ An important modification of lentiviral vectors is the change of the envelope protein from HIV-1, gp120, which is specific for the CD4 receptor, for VSV-G, a glycoprotein that recognizes a much more universal receptor across cell types called LDL-R.^26^ However, lentiviral vectors featuring VSV-G are unable to infect unstimulated T cells, as they lack the LDL-R.^27^

The efficiency of transduction of the CAR gene into T cells depends on the multiplicity of infection (MOI), which is the number of transducing lentiviral particles per cell.^28^ The higher the MOI, the more efficient the transduction. However, high MOI can result in tonic signaling, which is an overactivation of T cells produced by CARs interacting with each other.^29^ To correctly determine the MOI, the concentration of a batch of lentiviral particles is experimentally determined.^30^ Although it has been reported that in a suspension system, the number of viral particles that reach a cell follows a Poisson distribution,^31^ the use of a different culture system, such as PEG–Hep hydrogels, might change this distribution by breaking the randomness of the events of viral particles and cells finding each other. It is worth mentioning that lentiviral vectors are replication incompetent.^32^

Heparin-containing scaffolds have been suggested to bind LV and increase gene expression. Specifically, Thomas and Shea^33^ modified poly(lactide-*co*-glycolide) (PLG) scaffolds with polysaccharides and studied the loading of the scaffolds with LV and its effect on cell transduction. Interestingly, they found a 4.4-fold increase in transgene expression in heparin-modified scaffolds compared to unmodified scaffolds. Moreover, while the LV was released from the PLG scaffold in 48 h, nearly 100% was retained in heparin-modified scaffolds. In a subsequent study, the immobilization of heparin and chitosan on PEG hydrogels was evaluated.^34^ In this study, the authors differentiated between cells in the scaffold and around it, observing an important difference in gene expression between them, in favor of the first. Also, they show how nanoparticles improved LV incorporation into PEG hydrogels. They hypothesized that the hydrogel concentrates the vector within the cell microenvironment, thus promoting cell binding, internalization, and gene expression.

In this work, we designed novel biohybrid PEG–Hep hydrogels with optimized mechanical and structural properties to resemble human lymph nodes and improve primary human CAR T cell manufacture. Compared to the few recently published potentially competing systems,^35,36^ the biohybrid composition of the proposed hydrogels enables easy tuning of their mechanical (*G*′ = 0.5–3.1 kPa) and structural (average pore sizes of 20–120 μm) properties, while also maintaining the biocompatibility and bioactivity characteristic of natural polymers. In this regard, heparin demonstrated to be an interesting hydrogel component for CAR transduction enhancement, as it exploits the virus natural mechanism to approach cells through its affinity to extracellular heparan sulfate.^37,38^ More specifically, enhanced (anti-CD19) CAR expression and cell proliferation were obtained with stiff and heparin-containing hydrogels, as experimentally demonstrated and explained through modeling. It is also worth highlighting that the proposed hydrogels are not functionalized with cytokines or other biomolecules, which in the future, could further improve their functionality.^39^ Finally, in this work, we focused on studying CD4+ T cells, as they have recently been identified as capable of producing MHC-II-mediated cytotoxicity and, while not as efficient as CD8+ T cells, they possess the advantages of combining cytotoxicity with chemokine secretion, circumventing typical MHC-I related tumor resistance, and an improved longer-term effect.^40−43^

## 2 Experimental Section

### 2.1 Materials

Heparin was purchased from Acros (Fisher Scientific, USA). Then, it was functionalized with maleimide following a previously described protocol.^44^ 4-arm thiol-terminated poly(ethylene oxide) (PEG-SH; *M*_n_ 10,000 g/mol) was obtained from Nanosoft Polymers (USA). An aqueous suspension containing 10% w/v poly(methyl methacrylate) (PMMA) beads with diameters of 78.3 ± 1.7 and 119.3 ± 2.1 μm were purchased from microParticles GmbH (Germany). CellTrace carboxyfluorescein diacetate succinimidyl ester (CFSE) cell proliferation kit, penicillin/streptomycin (P/S), fetal bovine serum (FBS), Dynabeads, streptavidin-allophycocyanin (APC) conjugate, Hoechst, and sterile nuclease-free H2O AM9906 were provided from Thermo Fisher (USA). Miltenyi Biotec GmbH (Germany) provided the CD4+ T cell isolation kit. Lymphoprep was purchased from Stemcell Technologies (Canada). The antihuman CD3 and CD4 antibodies labeled with FITC and PE, respectively, and their controls used for flow cytometry were acquired from Immunotools GmbH (Germany). Anti-HIV1 p24 goat and antigoat H&L Alexa Fluor 488 antibodies were acquired from Abcam (UK). LightCycler 480 SYBR Green I Master and 384-well plates were purchased from Roche (Germany), enzyme restriction NotI-HF from New England Biolabs (USA), and DNeasy Blood & Tissue Kit and QIAquick Gel Extraction Kit from Qiagen (Germany). 1-Hydroxybenzotriazole hydrate (HOBT), *N*-(2-aminoethyl)maleimide trifluoroacetate salt (AEM), *N*-(3-dimethylamino-propyl)-*N*-ethylcarbodiimide hydrochloride (EDC·HCl), 2-(*N*-morpholino)ethanesulfonic acid (MES), Roswell Park Memorial Institute (RPMI)-1640 cell culture media, Dulbecco’s phosphate buffered saline (PBS), and any other products not specified here were purchased from Merck (USA).

### 2.2 Hydrogel Preparation

Bulk and IOPAL PEG–Hep hydrogels were prepared following previously reported protocols.^39,45^ The starting components used were nonfractioned heparin functionalized with maleimide (Hep–Mal) and commercial 10 kDa 4-arm PEG–SH. PEG–Hep hydrogels were cross-linked following a Michael reaction between Hep–Mal and PEG–SH in a molar ratio of 1.5:1 in PBS or cell media. The hydrogels were prepared in 5 mm diameter wells of a homemade Teflon template and had a volume of 30 μL. For bulk hydrogels, the mixture was directly added to the wells and incubated at 37 °C at least overnight resulting in a covalently cross-linked hydrogel. For IOPAL hydrogel formation, 100 μL of a PMMA beads aqueous suspension (10% w/v, non-cross-linked) with diameters of 78.7 ± 1.7 or 119.3 ± 2.1 μm were deposited into 5 mm wells and left to evaporate for at least 24 h. Once the opal was formed, the mixture of hydrogel components was carefully added to the wells, on top of the opal, and left to infiltrate the structure. Incubation was held at 37 °C for at least 2 days. Then, the hydrogel containing the opal was removed from the template and introduced in glacial acetic acid for at least 48 h (40 °C, 150 rpm; orbital shaker) to dissolve the PMMA beads of the opal. Finally, the acid was removed from the IOPAL hydrogels by washing them 3 times in PBS.

PEG–PEG hydrogels were prepared in the same Teflon template by mixing 2 kDa Mal–PEG–Mal and 2 kDa 4-arm PEG–SH at a 2:1 molar ratio. The mixture creates a hydrogel in a few minutes.

### 2.3 Hydrogel Characterization

A FEI Quanta 650F Environmental scanning electron microscope (ESEM; Thermo Fisher Scientific, USA) was used to image hydrated hydrogels. While SEM is usually thought for dry conductive samples, in this case a protocol was optimized for the imaging of hydrated hydrogels. More specifically, a temperature of 5 °C was used, and the pressure was abruptly dropped from 900 to 100–200 Pa in order to evaporate the water from the pores and expose the hydrogel structure. To characterize hydrogel stiffness, the storage modulus (*G*′) of the different hydrogels was determined using a small-amplitude oscillatory shear (SAOS) technique. *G*′ can be obtained from the linear plateau observed at low frequencies of frequency sweeps.^46^ First, strain sweeps were performed in order to localize the linear-viscoelastic region of each sample and choose an appropriate strain for the subsequent frequency sweeps. The experiments were performed at 37 °C. A Rheometer HAAKE RheoStress RS600 (Thermo Electron Corporation, USA) equipped with a 10 mm diameter rotor was used for all rheology experiments.

### 2.4 Lentivirus Preparation

The anti-CD19 CAR cloning and LV production of ARI-0001 was performed as previously described.^47,48^ Briefly, the variable light and heavy regions of the CAR were extracted from A3B1 hybridoma cells (A3B1 scFv). The CD8 hinge and transmembrane regions and the intracellular domains were extracted from 4-IBB and CD3z. The complete CAR sequence was cloned into a third-generation lentiviral vector. Lentiviral particles were produced by transfecting HEK293T cells with the transfer vector, packaging plasmids, and envelope plasmid using polyethylenimine (PEI) following the established protocols. The LV titration was also conducted following standard procedures. In brief, the number of transducing units per volume unit (TU/mL) was determined by the limiting dilution method. In summary, serial 1:3 dilutions of the viral particles were added to Jurkat cells in complete RPMI media. Cells were stained 72 h later with an antimouse immunoglobulin G antibody. Flow cytometry was used to analyze the results. The viral titer was calculated accounting for a dilution corresponding to 2–20% of positive cells.

### 2.5 CAR T Cell Culture

Buffy coats from healthy adult donors were supplied by “Banc de Sang i Teixits” (Barcelona, Spain). Permission for using such samples was obtained from the Ethics Committee on Animal and Human Experimentation of the Autonomous University of Barcelona (no. 5099). A previously established protocol based on a density gradient centrifugation and a magnetic selection was followed to obtain primary human CD4+ T cells.^39,45^ Only cell populations that were >90% (usually >95%) CD3 and CD4 positive were employed. After isolation, 100 μL of a suspension of 10^6^ CD4+ T cells/mL in supplemented RPMI cell media (10^5^ cells per well) were seeded on 96-well plates, either in suspension or on top of bulk or IOPAL hydrogels. Dynabeads were added at a 1:1 ratio (1 bead per cell). The cell culture was kept in the incubator at 37 °C and 5% CO_2_.

One day after the CD4+ T cell seeding, cells were transduced with a LV vector. First, the amount of LV to add per well was calculated according to the LV batch titer and the desired MOI, following eq 1. The calculated amount of LV was added to each well diluted in RPMI supplemented cell media to achieve a volume per well of 10 μL.
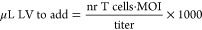
1

100 μL of additional RPMI supplemented cell medium were added to each well 2 days after the cell seeding. On day 5, the cultured CD4+ CAR T cells were collected from the wells by vigorous pipetting, recovering around 50% of the cells (Figure S1). Once collected, cells were separated from Dynabeads using a magnet. Optionally, the CAR T cell cultures were continued until day 9 by counting them every day and readjusting their concentration at 1 M/mL on day 5 and at 0.7–0.8 M/mL from day 6 on-wards.

### 2.6 CAR T Cell Proliferation and CAR Expression

To assess proliferation, CD4+ CAR T cells were stained with a CFSE cell proliferation kit after their purification and before seeding. The process was performed as indicated by the manufacturer’s instructions. On day 5 after seeding, debeaded cells were washed with PBS with 0.1% FBS and analyzed by flow cytometry. In some cases, the donor-to-donor variability was minimized by normalizing the results to the positive control of each donor. The data without normalization has also been included to the Supporting Information. For CAR expression analysis, CAR T cells were cultured until days 5 or 9, thenwere washed, and subsequently incubated first with a biotinylated anti-CAR antibody and then with a streptavidin-APC secondary antibody.^49^

### 2.7 Flow Cytometry

A CytoFLEX LX (Beckman Coulter, USA) equipment was used for all flow cytometry experiments, and the data was analyzed with the FlowJo software.

### 2.8 Real-Time Quantitative PCR (qPCR)

For gene quantification, CD4+ CAR T cells were expanded until day 5, and then the cells were collected from the wells, debeaded, and washed with PBS. After centrifugation, dry pellets were stored at −80 °C.

For the qPCR experiment, a LightCycler 480 Instrument was used. All samples and standards were run in duplicates, and with a negative control containing water instead of sample, also in duplicates.

For each one of the tested plasmids, a standard curve was prepared with number of copies per μL of: 10^8^, 10^7^, 10^6^, 10^5^, 10^4^, 10^3^, 10^2^. To prepare the standard, 5 μg of plasmid were digested overnight at 37 °C with the Notl restriction enzyme. The resulting product was run in an agarose gel and purified using the QIAquick Gel Extraction Kit. DNA concentration was tested using a NanoDrop 1000 spectrophotometer (Thermo Scientific, USA) and adjusted for the preparation of the standard curve.

The DNA of the samples to be analyzed was extracted using the DNeasy Blood & Tissue Kit (Qiagen). The DNA concentration was also tested with the NanoDrop spectrophotometer and adjusted to 100 ng/mL when possible, or otherwise to 25 ng/mL. Two genes (in duplicates) were tested for each sample. The GATA2 gene was used to normalize the results. The WPRE gene was used to quantify the ARI-001 anti-CD19 CAR presence. In each well of a 384-well plate, we added 3 μL H_2_O, 5 μL of LightCycler 480 SYBR Green I Master (2×), 0.5 μL of primer F, 0.5 μL of primer R, and 1 μL of DNA sample. The well plate was sealed, centrifuged, and kept in the dark until the experiment was run.

The thermocycler protocol consisted of 5 min at 95 °C for the first step followed by 40 cycles of: 95 °C for 10 s, 58 °C for 10 s, and 72 °C for 5 s, followed by 5 s at 95 °C, 1 min at 65 °C, and then 97 °C until the protocol ending.

The standard curve was analyzed, and the most diluted point eliminated whenever linearity was not maintained. The analysis of the melting curve was performed to make sure a single peak corresponding to a single amplicon was obtained for each sample.

Finally, the data was analyzed to obtain the number of copies per genome of the WPRE gene, which equals the number of copies of the CAR in each genome (eq 2). To achieve this, the number of copies of the samples were obtained by interpolating the results in the standard curves. Then, the GATA2 gene, which has 2 copies per genome, was used to normalize (eq 3).
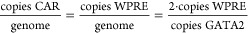
2
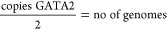
3

### 2.9 Computer Simulations

We have performed electrostatic potential and docking calculations and molecular dynamics (MD) simulations of a VSV-G lentiviral protein and heparin. Preparation and analysis of structures and trajectories was done with VMD^50^ and Chimera^51^ softwares.

The VSV-G lentiviral protein structure used in all the calculations was obtained from the Protein Data Bank database (code PDB: 5I2S) and it was protonated at pH = 7 using the PDB Reader & Manipulator of CHARMM-GUI.^52,53^ Electrostatic potential calculations were made at the nonlinear Poisson–Boltzmann (PB) level of theory using the APBS 3.4.1 software.^54^ Docking simulations between heparin and a VSV-G protein were performed using ClusPro 2 software.^55,56^ We considered a standard heparin fragment, a tetrasaccharide. The configurations predicted by docking were further refined by using them as initial configurations in MD performed with the NAMD 2.14 software.^57^ All simulations were done at *T* = 298 K in explicit water and RPMI buffer. The force field employed in the simulations is CHARMM36,^58^ which includes an appropriate parametrization for the protein and the heparin molecule. Full details of all calculations are provided in the Supporting Information. Our GitHub repository also provides open access to data from calculations, including structure files, and sample scripts.^59^

## 3 Results and Discussion

### 3.1 Synthesis of Hydrogels

**Figure 1:**
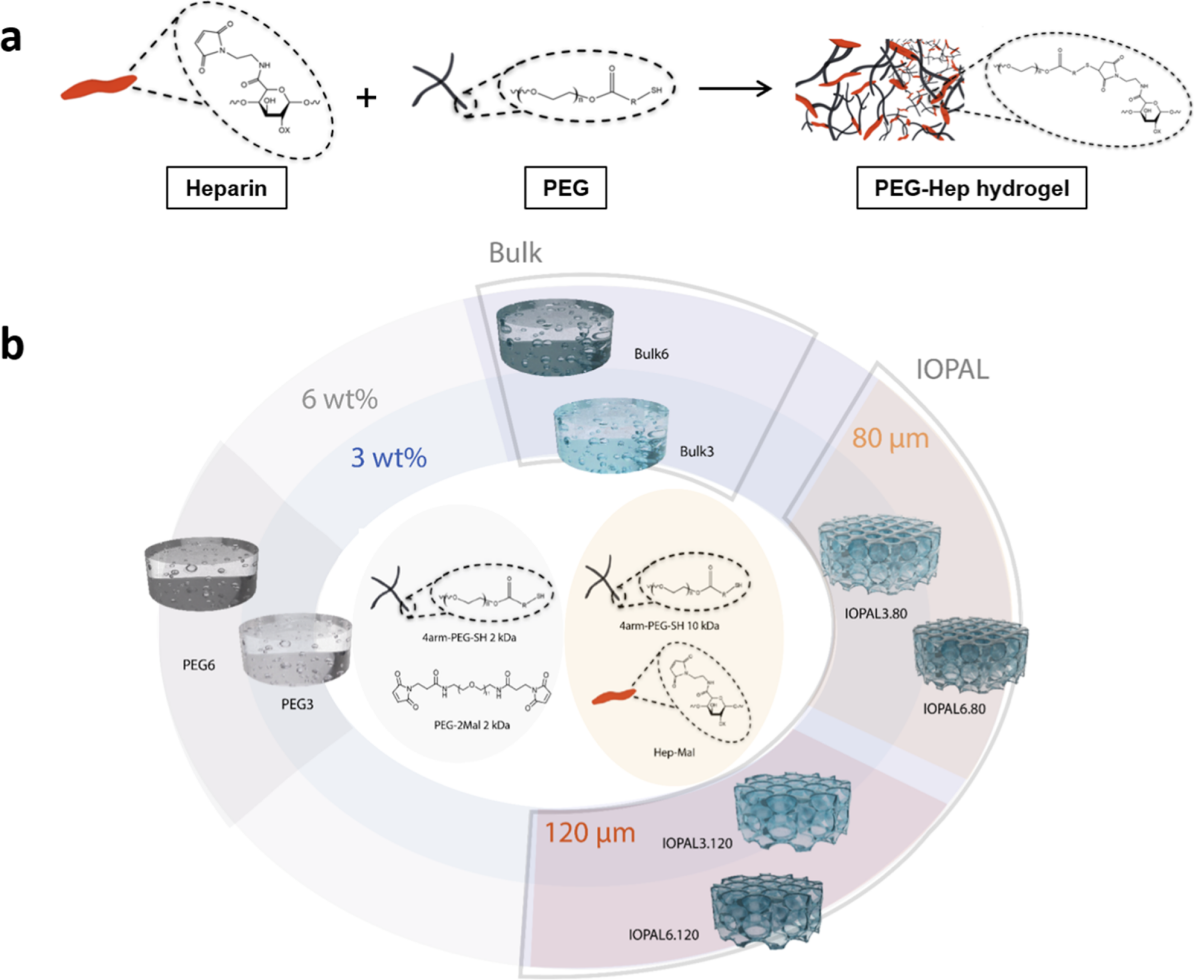
Hydrogels engineered for the culture of CAR T cells. (a) Synthesis of PEG–Hep hydrogels by click chemistry. Adapted with permission.^39^ Available under a CC-BY license. Copyright 2021 the authors. (b) Scheme showing the various hydrogel formulations produced and their nomenclature.

PEG–Hep hydrogels, in its bulk or IOPAL forms, have been shown to improve the expansion of CD4+ T cells by providing adequate mechanical and biochemical stimuli to the cells.^39,45^ Here, a new generation of PEG–Hep hydrogels was designed, synthesized, and characterized to dissect the impact of key physicochemical parameters, namely the chemical composition, stiffness, and structure (pore size and interconnectivity), on the culture of primary human CD4+ T cells transduced with a CAR against CD19, i.e., primary human CD4+ CAR T cells (Figure 1).

**Figure 2:**
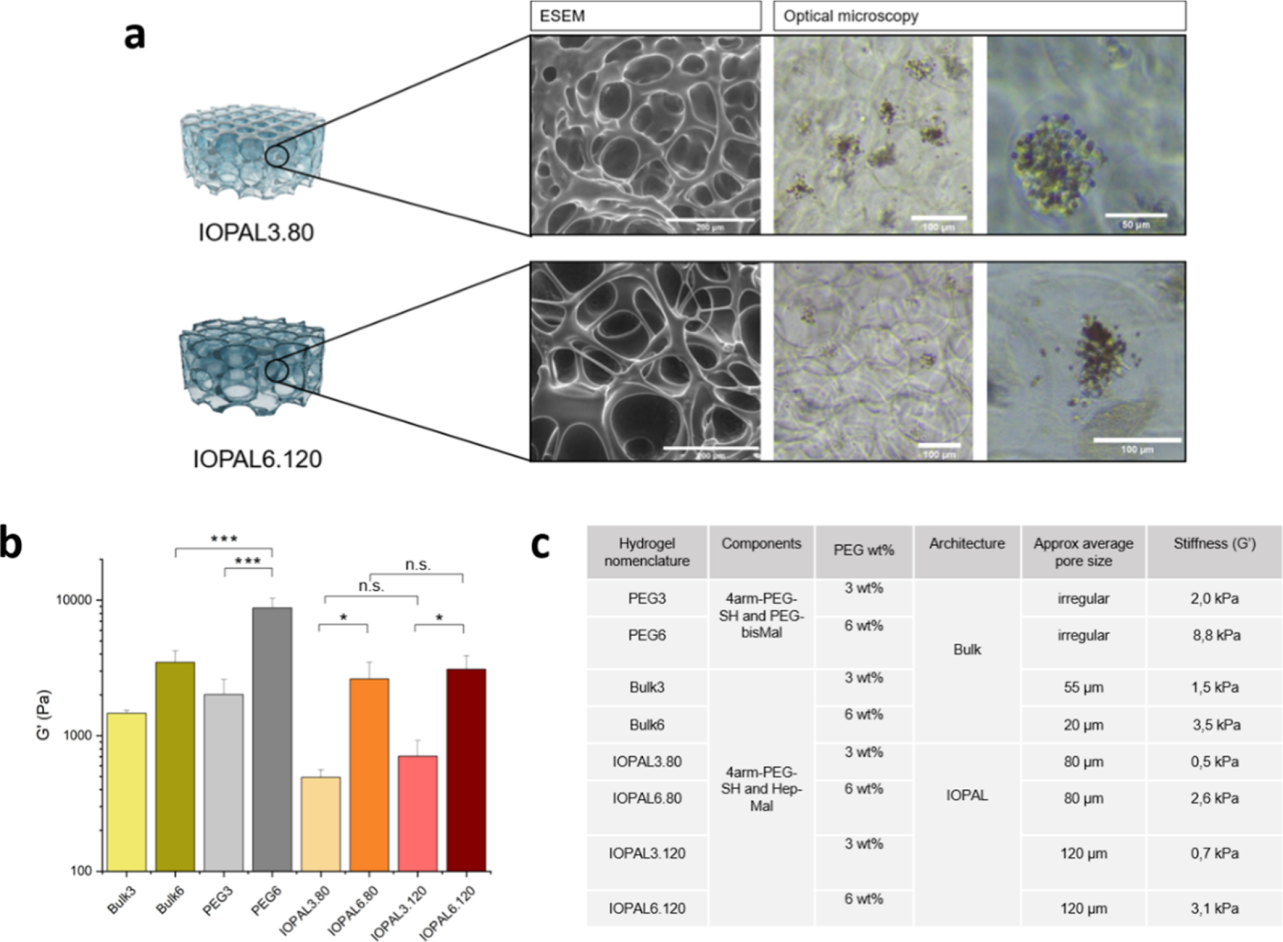
Physicochemical characteristics of hydrogels engineered for the culture of CAR T cells. (a) ESEM and optical microscopy images showing the porosity of IOPAL hydrogels and the presence of cell clusters inside the pores. (b) Storage modulus (*G*′) of the synthesized hydrogels (*N* = 2). *p < 0.05, ***p < 0.001 significance using a one-way ANOVA-Tukey’s multiple comparisons test. (c) Summary table of the physicochemical properties of the hydrogels studied for CAR T cell culture.

PEG–Hep hydrogels were prepared using thiolated 4-arm-PEG and maleimide-functionalized unfractionated heparin (Figure 1a). First, new IOPAL hydrogels with a pore size 120 μm were prepared (IOPAL3.120), enlarging the pore size of the previously described hydrogels, which featured pores of 80 μm (IOPAL3.80). Two more new types of IOPAL hydrogels were created with a higher weight percentage (wt %) than the reported one of 3 wt %. In particular, 6 wt % PEG was used (IOPAL6.80 and IOPAL6.120). Additionally, we prepared bulk hydrogels with the standard 3 wt % PEG (Bulk3) and the stiffer version with 6 wt % PEG (Bulk6). Finally, PEG–PEG hydrogels were prepared from 4-arm PEG–SH and Mal-PEG–Mal, i.e., hydrogels that do not contain heparin, in the two concentration forms (PEG3 and PEG6). By preparing all the mentioned combinations, a total of 8 different types of hydrogels were analyzed (Figures 1b and 2c).

### 3.2 Characterization of Hydrogels

First, the pore structure of hydrogels was explored by ESEM (Figure 2a). With this technique, we confirmed that the IOPAL hydrogels have a narrow pore size distribution with pores of sizes around that of the porogens used, which are PMMA beads of ca. 80 and 120 μm. Bulk hydrogels, unlike IOPALs, have only the intrinsic material porosity, which show pores of around 55 μm for Bulk3 and 20 μm for Bulk6 (Figure S2).^39^ Finally, PEG hydrogels show a more irregular microstructure, with parts containing no visible pores combined with others of irregular porosity (Figure S2).

It is also worth mentioning that the pores of IOPAL hydrogels can already be visualized with an optical microscope (Figure S3). During cell culture, clusters of primary human CD4+ CAR T cells form inside the pores of the hydrogels (Figure 2a). This compartmentalization reminds that of lymph nodes, in which T and B cells are separated in different zones, and cells cluster around dendritic cells.^60^

In the next step, the mechanical properties of the prepared hydrogels were determined by SAOS rheology measurements at 37 °C. In particular, the frequency sweeps were performed at a constant strain of 10 Pa and frequencies ranging from 0.1 to 15 Hz (Figures 2b and S4). These conditions ensured that all hydrogels were tested within their linear viscoelastic regime. PEG–Hep hydrogels with 3 wt % PEG showed *G*′ of 1.5 ± 0.1 kPa for Bulk3, 0.5 ± 0.1 kPa for IOPAL3.80, and 0.7 ± 0.2 kPa for IOPAL3.120. The difference between the bulk and IOPAL hydrogels may be accounted by the fact that IOPAL hydrogels have better interconnected pore structures (and larger pore sizes). On the other hand, PEG–Hep hydrogels with 6 wt % PEG showed *G*′ of 3.5 ± 0.7 kPa for Bulk6, 2.6 ± 0.9 for IOPAL6.80, and 3.1 ± 0.8 kPa for IOPAL6.120. As expected, *G*′ was lower when hydrogels were prepared with a 3 wt % PEG compared to those prepared with a 6 wt %. Finally, PEG3 and PEG6 hydrogels, which were prepared only with PEG reactants of short chains (2 kDa vs > 10 kDa of PEG–Hep hydrogels), presented higher stiffness. In particular, they presented *G*′ of 2.0 ± 0.6 kPa for PEG3 and 8.8 ± 1.6 kPa for PEG6. The properties of all tested hydrogels are summarized in Figure 2c.

### 3.3 Primary Human CD4+ CAR T Expansion and CAR Expression

Primary human CD4+ CAR T cells stained with CFSE were expanded in Bulk3 and IOPAL3.80 hydrogels, as well as in suspension with Dynabeads. These hydrogels were chosen as they have already demonstrated their capacity to improve CD4+ T cell culture of nontransduced cells.^39,45^ At day 5, cells were collected and the proliferation indexes were assessed by flow cytometry.^61^ Cell expansion was continued in suspension until day 9, in which CAR expression was also assessed by flow cytometry.

**Figure 3:**
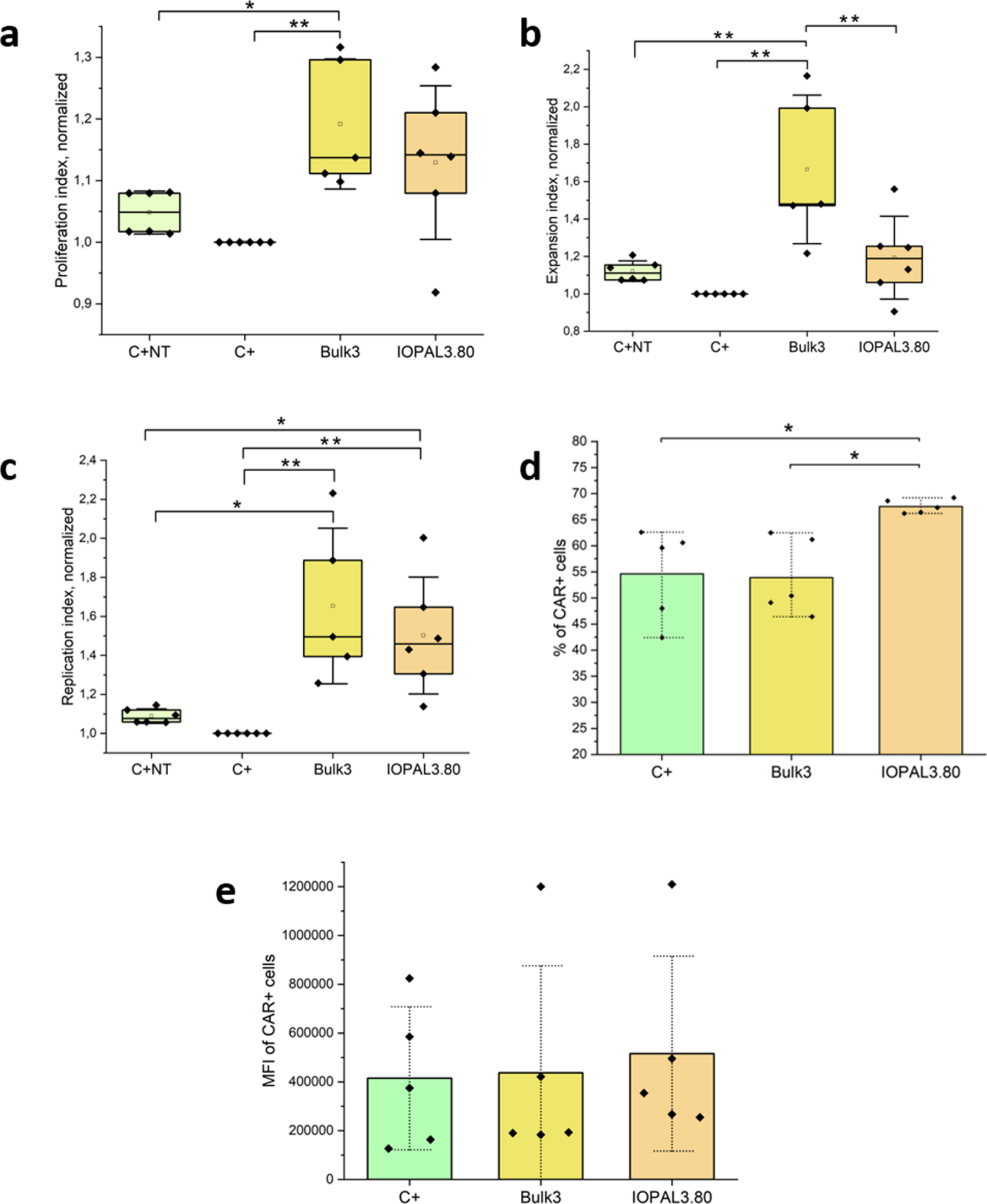
Primary human CD4+ CAR T cell cultures in suspension, bulk, and IOPAL hydrogels (*N*_donors_ = 5–6). The proliferation of primary human CD4+ CAR T cells was characterized on day 5 by flow cytometry and the data was normalized to the suspension culture (C+): (a) proliferation index, (b) expansion index, (c) replication index. CAR expression was also characterized on day 9 by flow cytometry: (d) percentage of CAR+ cells and (e) median fluorescence intensity (MFI). *p < 0.05, **p < 0.01 significance using a one-way ANOVA-Tukey’s multiple comparisons test.

A significant enhancement of the proliferation indexes was found when cells were seeded on both Bulk3 and IOPAL3.80 hydrogels compared to cells seeded in suspension (Figures 3a–c and S5). Specifically, cells cultured in Bulk3 and IOPAL3.80 hydrogels showed replication indexes 60% and 50% higher than those of cells expanded in suspension, in line with the previously reported results for nontransduced T cells.^39,45^ However, a subtle difference was observed between transduced and nontransduced cells expanded in suspension (10% higher replication index for nontransduced cells), which may account for the tonic signaling produced by CARs.^29^ In summary, our findings confirm that cells exhibit a preference for a tridimensional (3D) microenvironment that allows interaction with the extracellular matrix, as opposed to being in suspension.

In the next step, CAR expression was analyzed, using an anti-CD19 CAR produced with the LV ARI-0001.^47,48^ The percentage of cells showing positive expression of the CAR (Figures 3d and S6) and the median fluorescence intensity (MFI; Figure 3e), which gives a measurement of the level of CAR expression in each cell,^62^ were analyzed. In this case, cells expanded in IOPAL3.80 hydrogels showed the highest percentage of CAR+ cells (around 67%), while cells expanded in suspension or in Bulk3 hydrogels showed a similar percentage of CAR+ cells of around 55%. No differences between conditions were detected in the MFI. The significant enhancement of the CAR expression in terms of the percentage of CAR+ cells observed in IOPAL3.80 hydrogels triggered a further investigation of the impact of the hydrogel characteristics. First, however, the impact of the MOI in our system was systematically studied.

### 3.4 Effect of MOI on CD4+ CAR T Proliferation and CAR Expression

**Figure 4:**
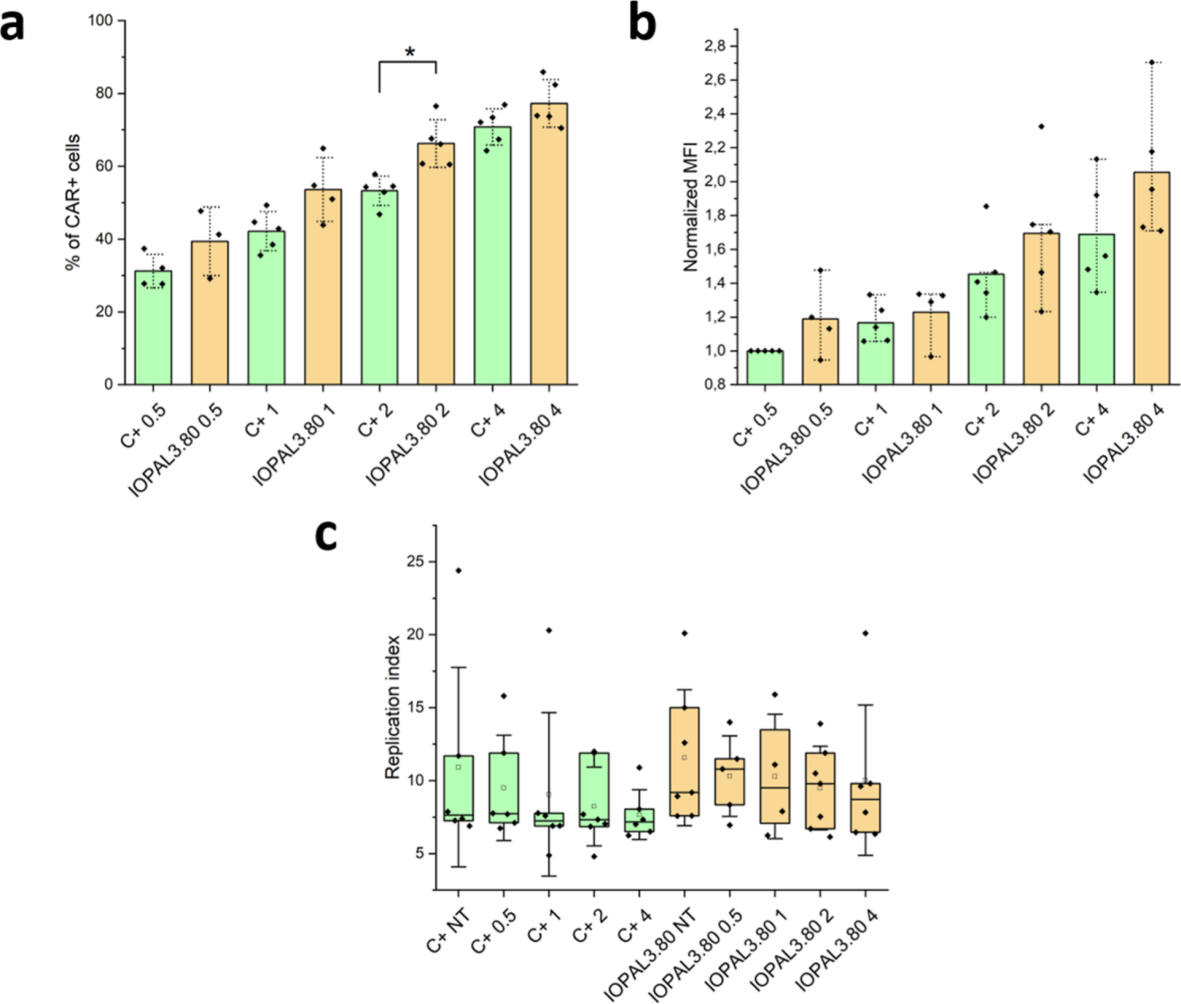
CAR expression and proliferation of primary human CD4+ T cells transduced with different amounts of LV (MOIs of 0.5, 1, 2, and 4) and cultured in suspension or in IOPAL hydrogels for 5 days (*N*_donors_ ≥ 4). (a) Percentage of CAR+ cells in each condition. (b) Normalized median fluorescence intensity (MFI) of the CAR+ cell population. (c) Replication index. *p < 0.05 significance using a one-way ANOVA-Tukey’s multiple comparisons test.

The MOI used in culturing CAR T cells has an impact on the characteristics of the resulting CAR T cellular product. The optimal value is a trade-off between high values prompt to tonic signaling and low values that could result in poor CAR expression. To examine this in the context of having a solid 3D microenvironment, which might disturb the standard Poisson distribution found in suspension, primary human CD4+ CAR T cells transduced with MOIs of 0.5, 1, 2, and 4 were cultured in both suspension and IOPAL3.80 conditions (Figure 4a).

CAR expression analysis on day 5 showed how increasing MOI results in a proportional increase of the % of CAR+ cells, as expected. For cells in suspension (C+), MOIs of 0.5, 1, 2, and 4 resulted in CAR+ % of 31, 42, 53, and 70, while percentages of 39, 53, 66, and 77 were obtained for cells cultured in IOPAL3.80 hydrogels. The difference is statistically significant in the case of MOI 2 (53% in suspension versus 66% in IOPAL3.80). Moreover, it is relevant to notice that the same CAR+ % was achieved using an MOI of 2 in suspension and an MOI of 1 in IOPAL3.80 hydrogels.

Cells cultured in IOPAL3.80 hydrogels not only show a higher CAR+ % for the same MOI, but also have a slight tendency to show a higher MFI (Figure 4b). In this case, however, there is no statistical difference between the IOPAL3.80 and suspension conditions.

The results obtained with the different MOIs are in line with previous studies,^28,63^ in which the transduction of the CAR depends on two main factors. First, the MOI, which determines the number of vector-cell encounters and second, the efficiency of transduction, which determines how many of those encounters end in transduction of the CAR, and it will depend on the cell type/lentiviral vector. As hypothesized, the presence of a hydrogel seems to disturb the Poisson distribution that applies in suspension by making cell-lentiviral vector encounters more probable.

CFSE-stained cells were analyzed by flow cytometry on day 5 (Figures 4c and S7). The results seem to show a slight decrease of the indexes as the MOI increases, especially for MOIs of 2 and 4, which is probably caused by tonic signaling. Also, cells cultured in IOPAL3.80 show higher proliferation, expansion, and replication indexes compared to cells cultured in suspension with the same MOI (enhancement of up to 20%). However, the differences were not found to be statistically significant. The improvement of the proliferation, expansion, and replication indexes when culturing nontransduced CD4+ T cells in IOPAL3.80 hydrogels had already been observed as mentioned before.^45^ Here, we confirmed that transduced cells with different MOIs show a similar behavior.

### 3.5 Impact of the Physicochemical Properties of Hydrogel Formulations on CAR Expression and Proliferation

**Figure 5:**
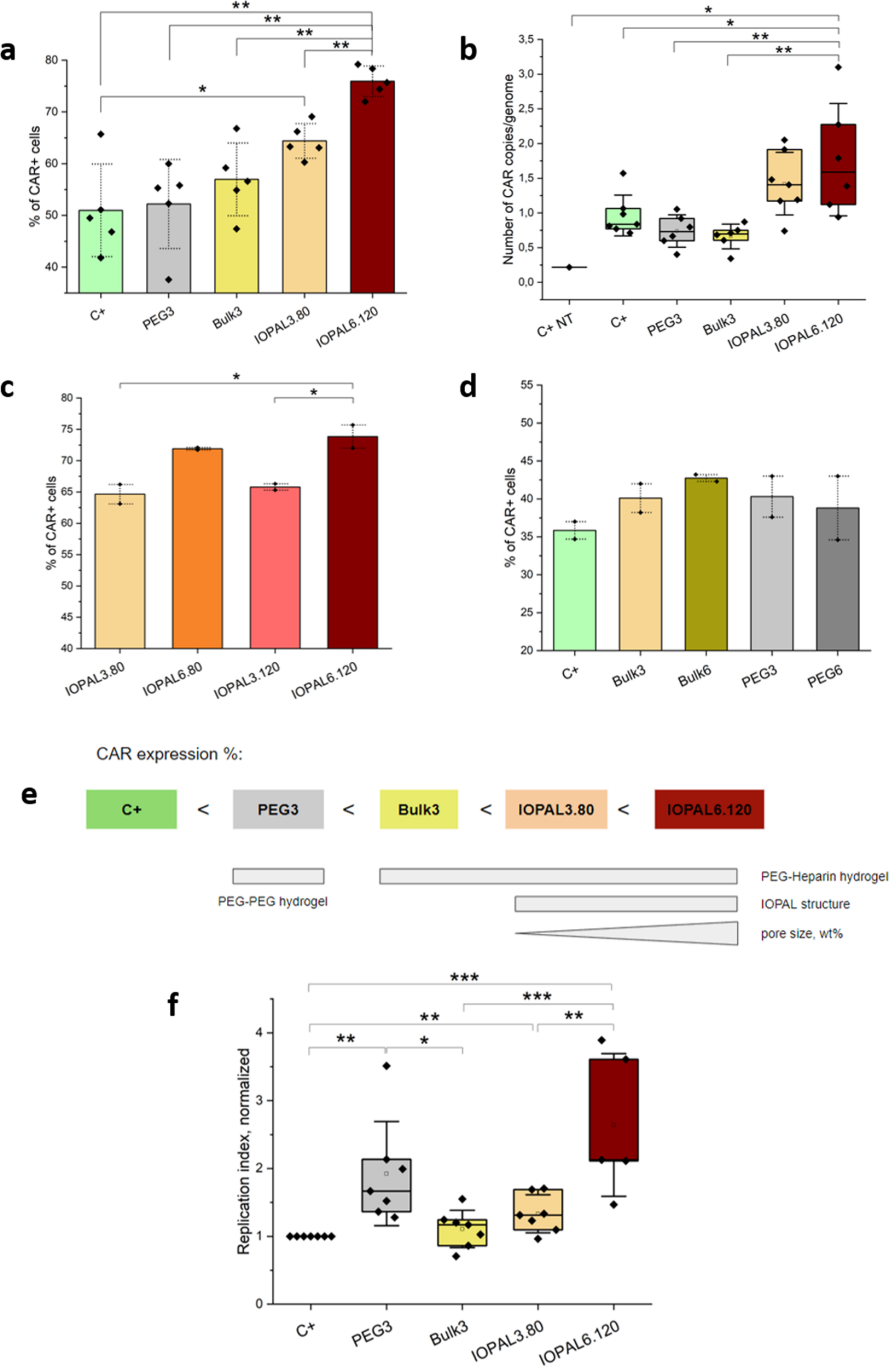
CAR expression and proliferation of primary human CD4+ CAR T cells cultured in different hydrogel formulations on day 5. (a) CAR expression profile (percentage of CAR+ cells over total alive cells) of CD4+ T cells transduced with the LV ARI-0001 at an MOI of 2 and cultured in suspension, PEG3, Bulk, IOPAL3.80, and IOPAL6.120 hydrogels (*N*_donors_ = 5). (b) Number of copies of the anti-CD19 CAR gene evaluated by RT-qPCR (*N*_donors_ = 5). (c) CAR expression of CD4+ CAR T cells cultured in IOPAL hydrogels with different stiffness and pore size (*N*_donors_ = 2). (d) CAR expression evaluated in bulk PEG–Hep and PEG–PEG hydrogels with different stiffness (*N*_donors_ = 2). (e) Scheme of the different physicochemical properties of the tested hydrogels and their relative position in their ability to promote CAR expression, from less to more % of CAR+ cells. (f) Replication index normalized to the C+ (*N*_donors_ = 5–6). *p < 0.05, **p < 0.01, ***p < 0.001 significance using a one-way ANOVA-Tukey’s multiple comparisons test.

CD4+ CAR T cells transduced with an MOI of 2 were cultured in hydrogels with different physicochemical parameters, with the aim of elucidating the impact of each parameter on CAR expression and proliferation (Figures 5 and S8).

The percentage of CAR+ cells found after 5 days of culture in four different hydrogels was analyzed (Figure 5a). These hydrogels are the previously described Bulk3 and IOPAL3.80 hydrogels, as well as the PEG3 hydrogels to assess the effect of the presence of heparin, and the IOPAL6.120 which feature the most extreme architecture with large pores and high stiffness. The percentage of CAR+ cells in suspension (C+) was found to be slightly over 50%, while in the nonheparin-containing PEG3 hydrogels was 55% and in the Bulk3 hydrogels 57%. The IOPAL3.80 showed a small increase with a percentage of CAR+ cells of around 64%. Interestingly, the IOPAL6.120 exhibited the highest (and statistically significant) CAR expression with 75% of CAR+ cells. All hydrogel conditions showed increased CAR expression compared to the C+, pointing to a positive effect of the tridimensionality of the hydrogels compared to suspension cultures, as previously shown.^39,45,64,65^

The reported interaction between heparin and LV, which can be explained by the natural viral affinity to heparan sulfate as cell-binding strategy,^66^ might also play a role. In this regard, the CAR expression of CD4+ T cells cultured in bulk hydrogels with or without heparin shows a slight increase when heparin is present. A plausible hypothesis is that both cells and LV are drawn toward the heparin-containing hydrogel structure, therefore increasing their potential encounter. The IOPAL structure, which should further increase the interactions between the two components due to its interconnected pores of a defined size, proved to be advantageous for CAR expression compared to homogeneous bulk hydrogels. However, the highest percentage of CAR+ was obtained with the IOPAL6.120 hydrogels, which not only contains the highest amount of heparin, but also the largest interconnected pores. Moreover, higher amounts of PEG/heparin result in higher stiffness.^39^

The same conditions were analyzed by real-time quantitative qPCR to determine the CAR sequence in the cells’ genome instead of the receptor on the cell membranes (Figure 5b). The experiment showed a median number of copies per genome of around 1 in the C+, Bulk3, and PEG3 conditions. In the IOPAL3.80 and IOPAL6.120, however, cells presented a higher number of gene copies, proving that the CAR transduction was more efficient when CD4+ T cells were cultured in IOPAL hydrogels compared to other hydrogels or in suspension. The differences were found statistically significant when comparing the IOPAL6.120 with all other conditions, except for the IOPAL3.80.

To shed light on the previous findings, the CAR expression of CD4+ CAR T cells cultured in IOPAL hydrogels with four possible combinations, arising from 3 or 6 wt % PEG and 80 or 120 μm porogen sizes, was studied by flow cytometry (Figure 5c). The percentage of CAR+ cells was 65% in IOPAL3.80 and 66% in IOPAL3.120, while it was 72% in IOPAL6.80 and 74% in IOPAL6.120. Thus, the pore size does not seem to be responsible for the differences observed in CAR expression. On the other hand, the matrix stiffness and/or amount of heparin seems to play a key role.

To further investigate this effect, bulk and PEG hydrogels also with a 3 and 6 wt % of PEG were evaluated (Figure 5d). In this case, the percentage of CAR+ cells was 40% in Bulk3 and 43% in Bulk6 hydrogels, while it was 41% in both PEG3 and PEG6 hydrogels. Although nonsignificant results were obtained, there is a slight improvement when moving from 3 to 6 wt % in bulk hydrogels, unlike the so-called PEG hydrogels, suggesting an effect of the heparin. It is worth noting that PEG hydrogels are stiffer than bulk hydrogels with the same proportion of PEG, as their components have shorter chains. In summary, the results seem to point to a synergy between heparin presence, IOPAL architecture, and appropriate stiffness for a positive modulation of the CAR expression of CD4+ CAR T cells (Figure 5e).

Additionally, the expansion of the CD4+ CAR T cells in the different hydrogel formulations was evaluated, as performed before (Figures 5f and S9). The trend observed was similar to the one of the CAR expression, with a significant improvement when cells were cultured in IOPAL6.120 hydrogels. However, the PEG3 hydrogels showed the second-best result, significantly improving CAR T proliferation indexes compared to C+ and Bulk3 conditions. The higher stiffness of PEG hydrogels could explain these results, as this could lead to an improved mechanotransduction that would favor activation, migration, and ultimately proliferation of T cells.^67^ Indeed, the importance of the forces that T cells can exert through the TCR toward their activation stimuli is well-known. The observed trend is that, in the range of physiological stiffness, which is around 0.5–100 kPa, stiffer substrates are preferred by T cells resulting in improved migration and stronger activation.^68−70^ These findings are in agreement with the results shown by PEG3 and IOPAL6.120 hydrogels, which are stiffer than Bulk3 and IOPAL3.80 hydrogels, and achieved higher primary human CD4+ CAR T proliferation.

Furthermore, there seems to be an influence of the IOPAL structure as well as the heparin content in cell proliferation, as the IOPAL6.120 hydrogels exhibited the highest proliferation. The IOPAL structure is characterized by a narrow pore size distribution and an excellent interconnectivity of the pores. This defined architecture allows a good infiltration and migration of T cells, and provides cavities for cells to cluster (Figure 2a), with a compartmentalization that may resemble that of lymph nodes.

### 3.6 Molecular Basis for the Interactions between Lentiviral Particles and Heparin

**Figure 6:**
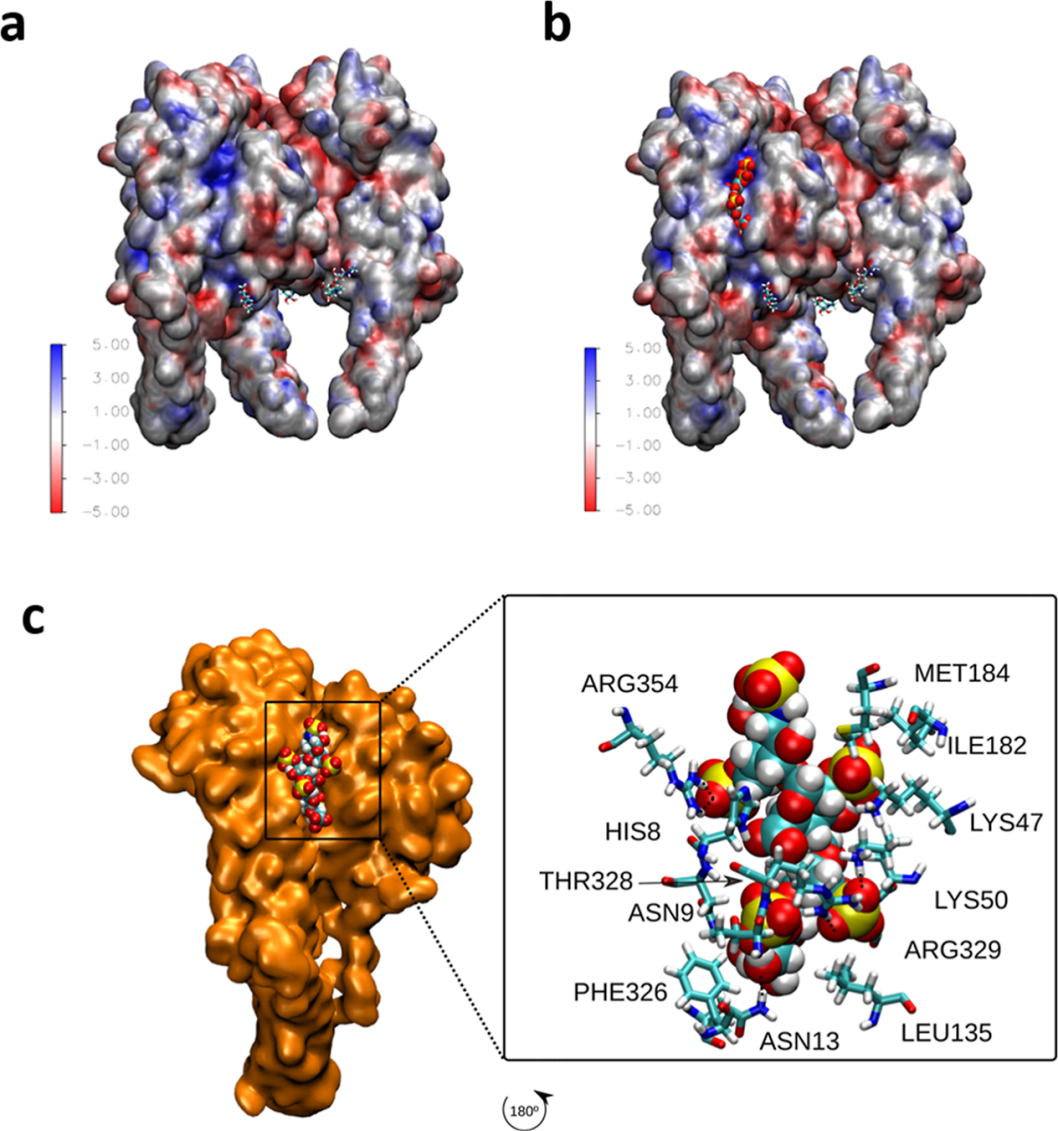
MD simulations exploring the interactions between lentiviral particles and heparin. (a) Electrostatic potential from PB calculations evaluated over the surface of the protein (in *kT*/e = 25 mV units). (b) Binding of heparin as identified by docking calculations (configuration with the largest number of contacts between the protein and heparin). (c) Snapshot from MD simulations showing an equilibrated configuration obtained from the structure in (b). The zoom shows the amino acids in contact with heparin (note the rotation of the structure in order to show the view from inside the protein). Image created with VMD.^50^

To further understand the system, the interactions between lentiviral particles and heparin was explored using MD simulations. More specifically, we studied the interactions between the VSV-G protein (responsible for the interaction of the lentiviral particles and the environment) and a heparin fragment (a dimer). Our electrostatic calculations (Figure 6a) show that the VSV-G protein has a region with a large positive electrostatic potential (≈125 mV) suitable for a strong electrostatic interaction with the negatively charged heparin. It should be noted that this region of the protein is the one interacting with the LDL receptor and therefore responsible for the interaction of the virus with cells (Figure S10).

Docking calculations indicate that heparin is able to attach electrostatically to this region, with several possible poses (see Figure 6b for the pose with the largest number of contacts with the protein and Figure S11 for further possible configurations).

The stability of the most probable configurations with heparin bound to VSV-G, as identified by docking calculations, was confirmed by MD simulations. We considered the configurations identified by docking as starting configurations for long MD runs (see Supporting Information for details).

The equilibrated configurations from MD are very close to the ones identified by docking calculations (see Figure 6c for the refinement of the structure of Figures 6b and S12 and S13 for other possible configurations). As seen in Figure 6c, in this equilibrated configuration the heparin fragment is bound to an average of ≈12 protein amino acids, corresponding to 6 amino acids per heparin monomer. The interaction is primarly electrostatic, with some contribution from hydrogen bonds (3.5 on average). In summary, our calculations confirm the expectation that the region with high positive electrostatic potential of the VSV-G protein is able to bind heparin. Therefore, theoretical calculations support the concept that the lentiviral particles bind electrostatically the hydrogels containing heparin.

## 4 Conclusions

We have shown that the manufacture of primary human CD4+ CAR T cells in engineered PEG–Hep hydrogels can positively modulate their proliferation and efficiency of CAR transduction, due to the presence of a 3D support, its microstructure, stiffness, and composition.

Further improvements to PEG–Hep hydrogels for CAR T cell expansion could be explored, such as the incorporation of homing chemokines, thus improving the simulation of the lymph node microenvironment.^39^ Moreover, longer culture times and functionality studies will also be needed to continue advancing toward the clinical application, in addition to recent scalability studies using 3D printing.^71^

In conclusion, this study enhances our fundamental understanding of the transduction step in CAR T cell fabrication and suggests modifications that could significantly advance the broader clinical implementation of CAR T therapy.

## Data Availability

The data that support the findings of this study are available from the corresponding author upon reasonable request.

## Supporting Information Available

It contains additional details on hydrogel characterization by ESEM, optical and confocal microscopy and rheology; further data on CAR expression and proliferation of CD4+ CAR T cells; and additional computer simulations of the interactions between VSV-G protein and heparin or LDL-R receptor (PDF)
