# Supplementary material for: Lymph-Node Inspired Hydrogels Enhance CAR Expression and Proliferation of CAR T Cells
Source: ACS Appl Mater Interfaces. 2025 Mar 5;17(11):16548–60. doi: 10.1021/acsami.4c19942 (PMC11931490; doi:10.1021/acsami.4c19942)
Supplement: Supplementary file 1 — am4c19942_si_001.pdf [file am4c19942_si_001.pdf]

Supporting Information

Lymph-node inspired hydrogels enhance CAR expression and proliferation of CAR T cells

Miquel Castellote-Borrell,[1] Marc Domingo,[2] Francesca Merlina,[1] Huixia Lu,[2,3] Salut Colell,[4] Mireia Bachiller,[4] Manel Juan,[4] Sonia Guedan,[4] Jordi Faraudo,[2,*] Judith Guasch[1,*]

[1]Dynamic Biomaterials for Cancer Immunotherapy, Max Planck Partner Group, Institut de Ciència de Materials de Barcelona (ICMAB-CSIC), Campus UAB, 08193 Bellaterra, Spain

[2]Soft Matter Theory Group, Institut de Ciència de Materials de Barcelona (ICMAB-CSIC), Campus UAB, 08193 Bellaterra, Spain

[3]Department of Physics, Universitat Politècnica de Catalunya-Barcelona Tech (UPC), 08034 Barcelona, Spain

[4]Department of Hematology, Hospital Clinic, Institut d'Investigacions Biomèdiques August Pi i Sunyer (IDIBAPS), 08036 Barcelona, Spain

E-mail: jfaraudo@icmab.es, jguasch@icmab.es

1. Percentage of cells eluted from the hydrogels

In this work, the recovery of cells from hydrogels was performed by simply pipetting the media on top of the hydrogels up and down vigorously, as enough cells could be recovered for experimentation. To determine though the cell recovery obtained, we compared the cell number obtained with a Neubauer chamber to that expected considering the expansion index[1] as obtained by CFSE staining and flow cytometry analysis of CD4+ T cells cultured for 5 days. As representative examples, suspension, Bulk3, and IOPAL3.80 hydrogels were used (Figure S1).

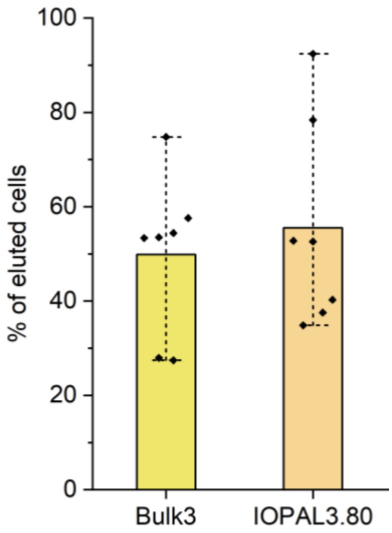

Figure S1. Percentage of eluted cells from Bulk3 and IOPAL3.80 PEG-Hep hydrogels 5 days after seeding (N = 7). The values, which were normalized to the suspension condition, were calculated by dividing the number of counted cells with a Neubauer chamber after elution by the expected cells using the expansion index coming from CFSE flow cytometry analysis.

The results have been normalized to the suspension condition (Control +), in which cells are expected to be easily collected. Thus, a 50±19% of cells were collected from Bulk3 hydrogels and a 56±27% in IOPAL3.80 hydrogels.

2. Environmental scanning electron microscopy (ESEM)

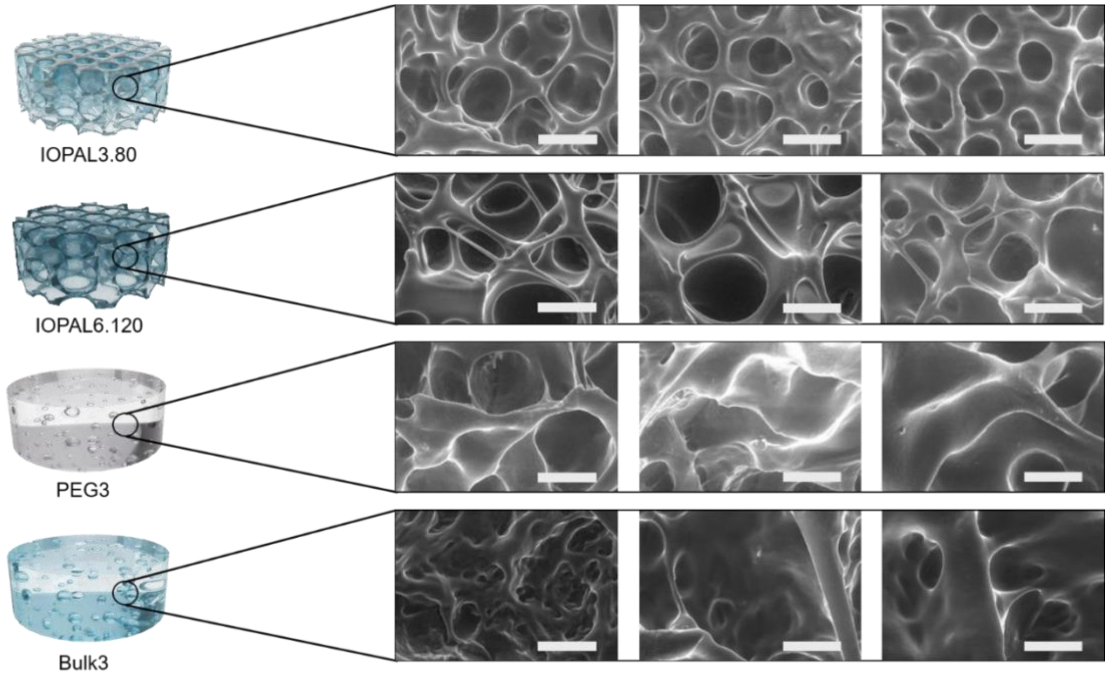

Figure S2. Schematic figure of different hydrogels and their corresponding ESEM images. Scale bar = 100 µm.

3. Optical and confocal microscopy

Optical microscopy captures were taken using an inverted microscope IM-3 (Optika Microscopes, Italy) and an attached Optikam PRO6 Digital Camera (optika Microscopes, Italy).

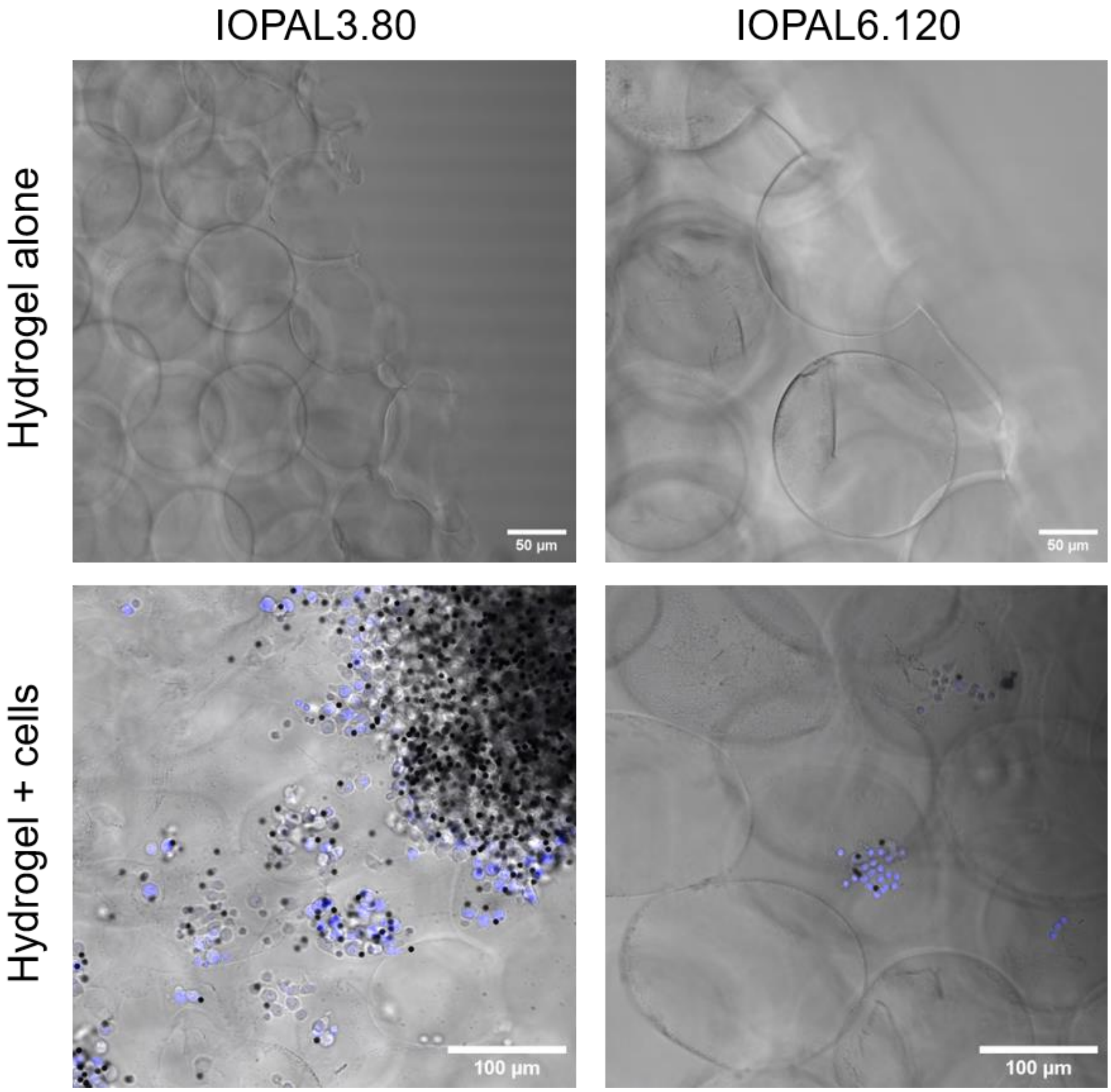

Figure S3. Confocal microscope images showing the bright field in gray and cell nuclei stained by Hoechst in blue. The IOPAL pores can be observed at the hydrogel border (upper captions). CD4+ T cells and Dynabeads inside the hydrogels (lower captions).

4. Rheology characterization. Frequency sweeps

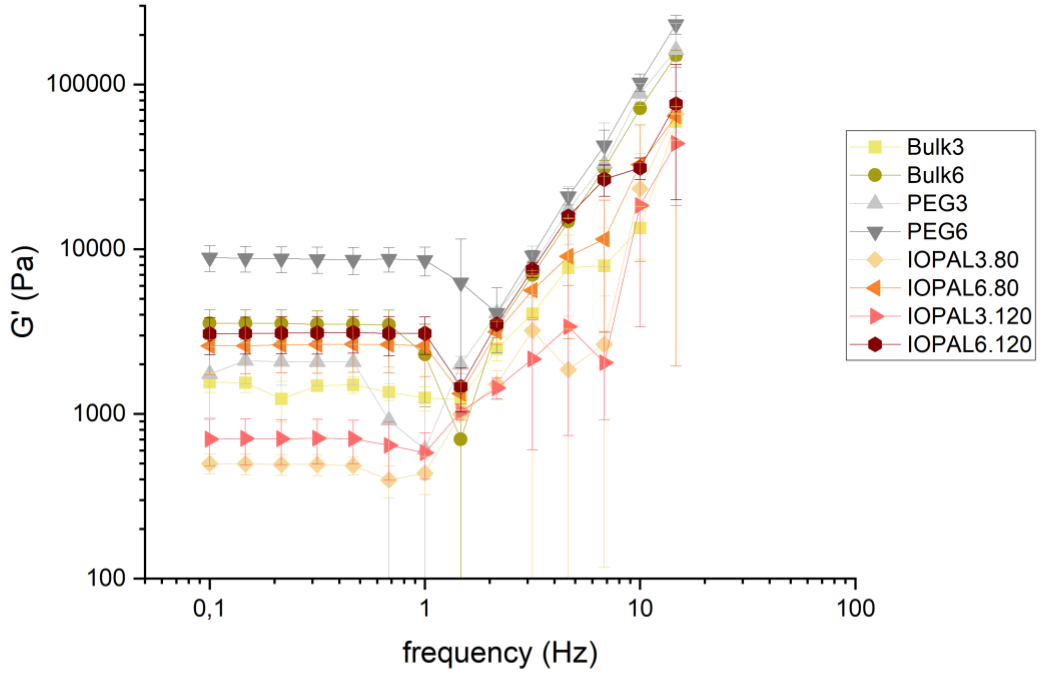

Figure S4. Storage modulus (G') of hydrogels at frequencies of 0.1 to 15 Hz and a constant strain of 10 Pa (N=3). The linear regime observed at lower frequencies corresponds to the equilibrium modulus (Ge).

5. Lentivirus ARI-001 characterization

To better understand the interaction between the LV used for CAR transduction and PEG-Hep hydrogels, we characterized the LV by DLS. Z-potential was found to be -5.63 mV, which would agree with the general knowledge of LV being negatively charged.

6. Proliferation of primary human CD4+ CAR T cell culture in suspension, Bulk and IOPAL hydrogels (without normalization)

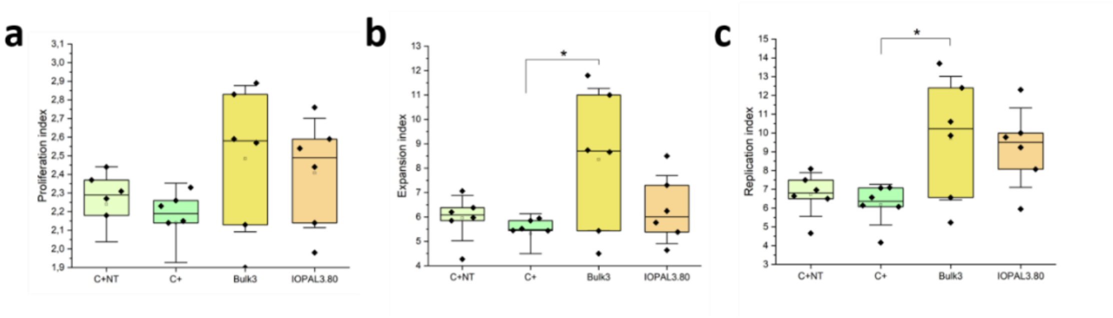

Figure S5. Proliferation of primary human CD4+ CAR T cells performed by flow cytometry on day 5 ($N_{donors} \geq 5$). a) Proliferation index, b) Expansion index, c) Replication index. * $p < 0.05$ significance using a one-way ANOVA - Tukey's multiple comparisons test.

7. CAR expression analysis by flow cytometry

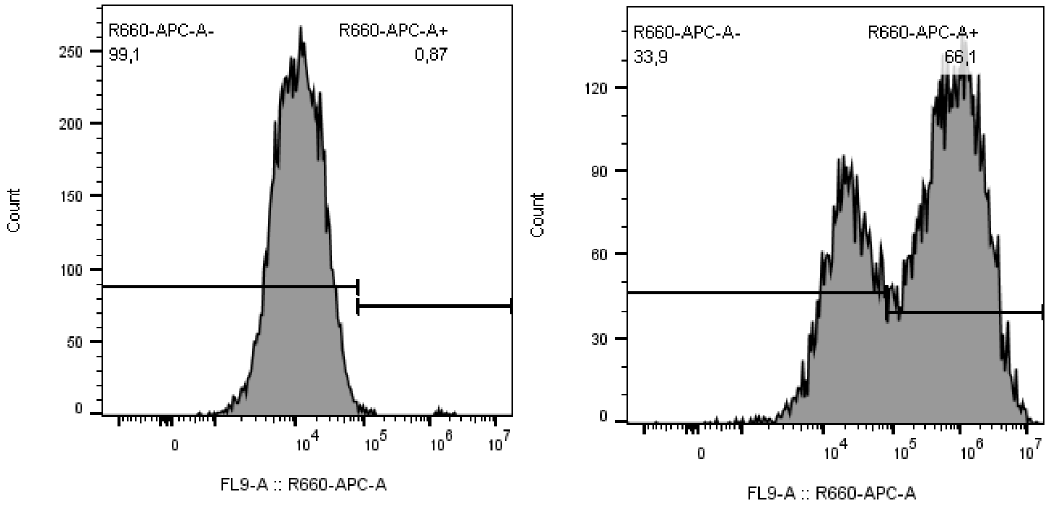

Figure S6. Representative APC histograms of non-transduced cells (left) and cells transduced with an MOI of 2 (right) cultured 5 days in IOPAL3.80 hydrogels.

8. Proliferation of CD4+ CAR T cells transduced with different MOIs and cultured in suspension or IOPAL hydrogels

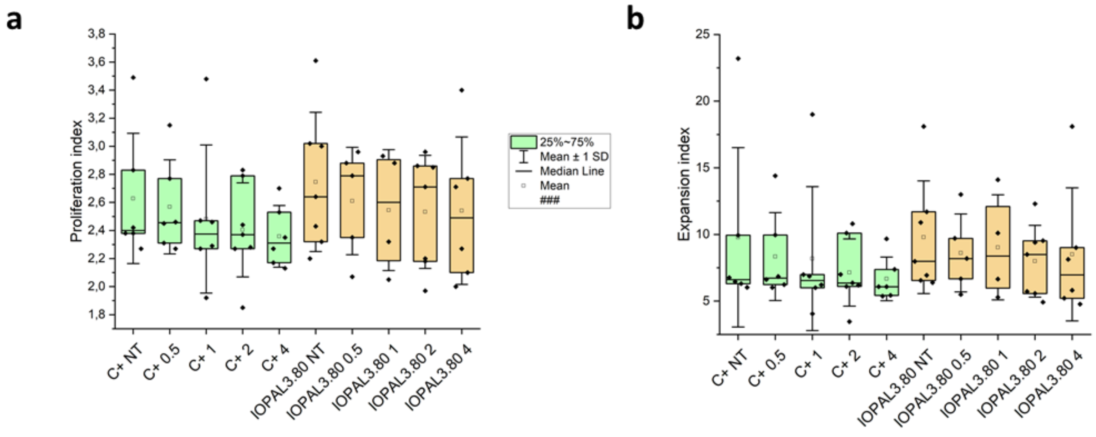

Figure S7. Proliferation of CD4+ T cells transduced with different amounts of LV (MOIs of 0.5, 1, 2 and 4) and cultured in suspension or in IOPAL3.80 hydrogels for 5 days ($N_{donors} \geq 4$). a) Proliferation index. b) Expansion index. Significance was assessed using a one-way ANOVA test.

9. Flow cytometry representative histograms of CAR expression and cell proliferation

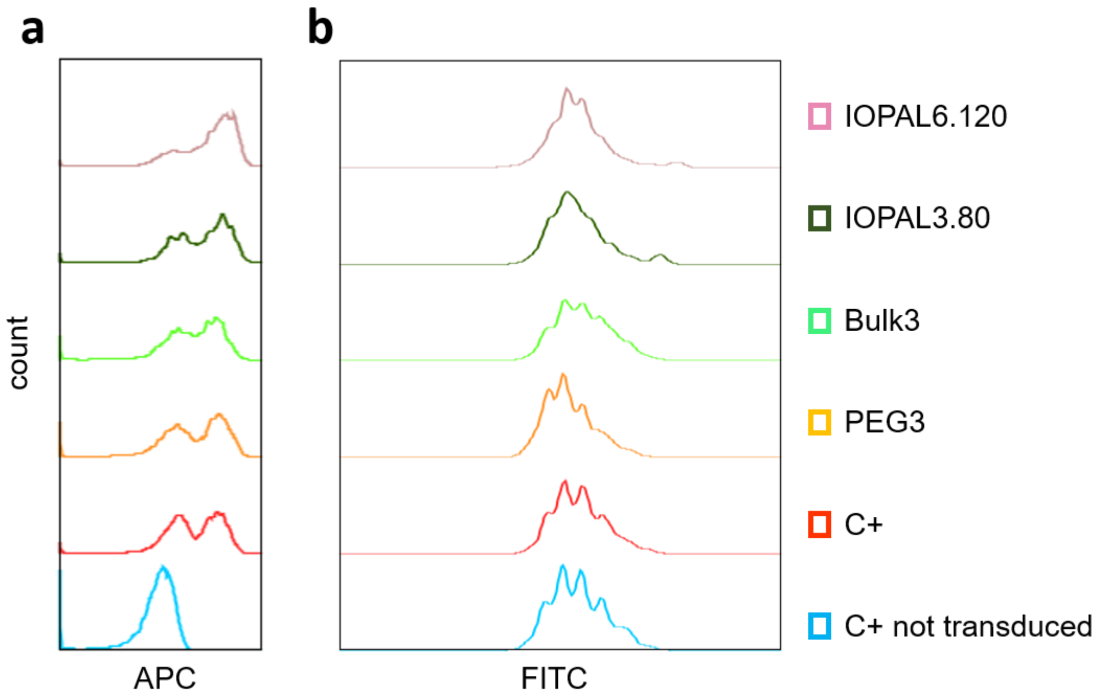

Figure S8. Flow cytometry representative histograms of CAR expression and proliferation on day 5. a) APC histograms of a representative donor showing two peaks: a first peak of CAR- cells and a second peak of CAR+ cells. b) FITC histograms showing the CFSE signal of each sample. Each peak corresponds to a generation of the expanded CAR T cells.

10. Proliferation of primary human CD4+ CAR T cells in various hydrogel formulations

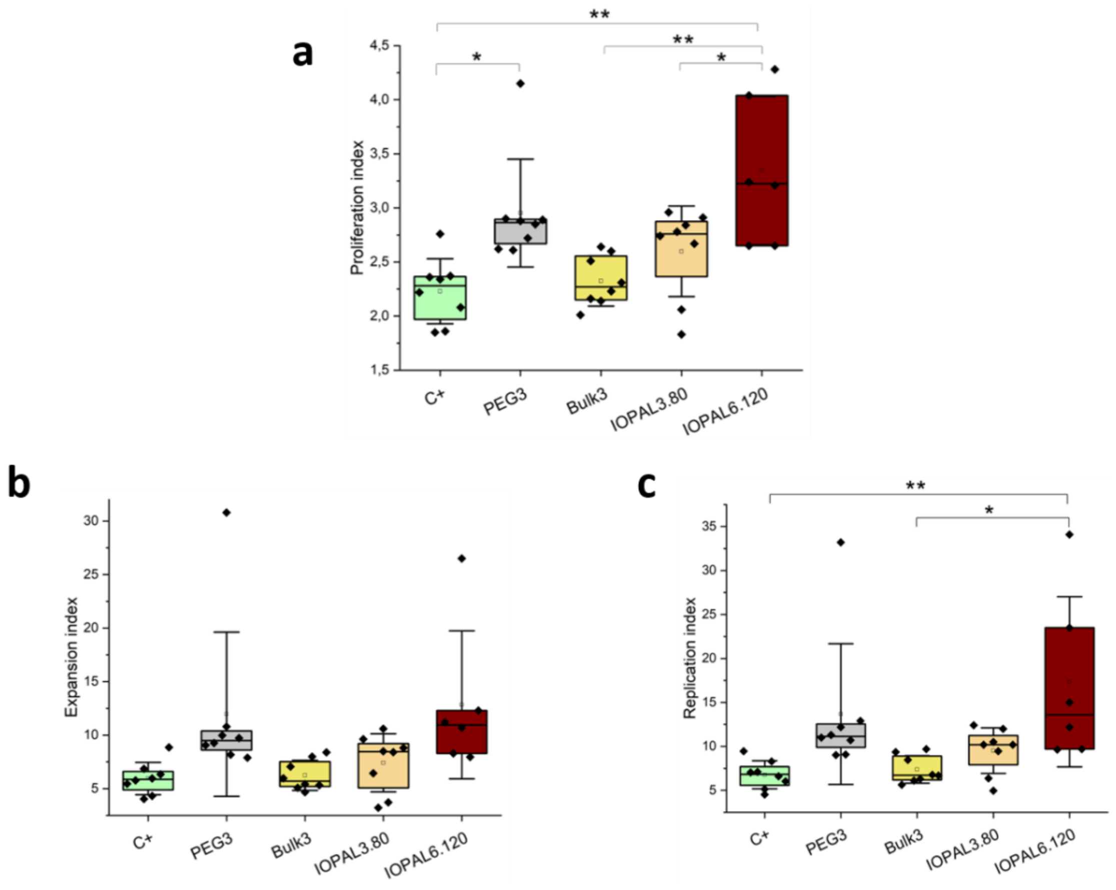

Figure S9. Proliferation of primary human CD4+ CAR T cells cultured in various hydrogel formulations on day 5 (N_{donors} = 6). a) Proliferation index. b) Expansion index. c) Replication index. * $p < 0.05$ and ** $p < 0.01$ significance using a one-way ANOVA - Tukey's multiple comparisons test.

11. Computer Simulations: full methods and detailed results

APBS Calculations. The electrostatic potential of the VSV-G protein was calculated using the online Poisson Boltzmann (PB) server with APBS 3.4.1 software.[2] The pqr input file of the VSV-G protein was generated using the APBS tool of VMD from the PDB atomic coordinates considering the CHARMM radii. The electrostatic potential was calculated using the full nonlinear PB theory (Figure S10).

Docking Simulations: Docking simulations of the VSV-G protein and heparin were performed using the ClusPro 2.0 server.[3] In the calculations, we considered the standard ClusPro heparin fragment (a tetrasaccharide). The protein glycans were not considered in the docking calculations since they are not modelled in ClusPro. The server generated a total of 10 models of a heparin fragment docked to the VSV-G protein, which correspond to the same binding site (the region of the protein with the highest electrostatic potential). The three models proposed as best models by the algorithm are shown in Figure S11, including the electrostatic potential map and a highlight of the protein residues interacting with the heparin.

Molecular Dynamics Simulations: Molecular dynamics (MD) simulations were performed by using NAMD 2.14 software (Figures S12-S13). The Newton equations of motion were solved numerically with a time-step of 2 fs. In all simulations the temperature was kept constant at 298 K using the Langevin thermostat with a relaxation constant of 1 ps^{-1}. The equations of motion were integrated with a 2 fs time step and the electrostatic interactions were updated every 4 fs. All bonds between heavy atoms and hydrogen atoms were kept rigid. In all the simulations we employed periodic boundary conditions in all directions. Lennard-Jones interactions were computed with a cutoff of 1.2 nm and the switching function starting at 1.0 nm. Electrostatic interactions were

computed using Particle Mesh Ewald (PME) algorithm using a real space cutoff set at 1.2 nm and a PME grid at 1.0 Å.

The best two heparin-protein complex structures obtained from the docking calculations were considered as initial structures for MD simulations. In both cases, the protocol was the following. Missing hydrogen atoms were added to the heparin fragments in the final ClusPro structures by using Chimera software. The complex protein-heparin was solvated in TIP3P water and the RPMI buffer was added (NaCl as a neutralizing salt). We performed a minimization of the system and an NPT equilibration run (~200 ns) afterwards. We employed a Nosé-Hoover isotropic barostat with an oscillation period of 100 fs and a damping time of 50 fs. The heparin molecule stays in the pocket found by ClusPro during this equilibration run. To accelerate the MD simulations, we selected the VSV-G protein monomer in which the heparin is attached (instead of the whole protein trimer) to perform further MD production runs. We solvated the system and added the RPMI buffer again. The reduced system is further minimized and equilibrated with a short (100 ps) NPT simulation (1 atm) to achieve the proper water density. Then, we run the two production simulations in the NVT ensemble. For both NVT simulations the heavy atoms from residues 60 to 88 were frozen along the simulation to keep the fusion loops rigid, mimicking the attachment of this protein in the virus envelope membrane (these fusion loops correspond to protein residues that are oriented towards the virus membrane). The characteristics parameters of both simulations are given in Table S1.

For both simulations reported in Table S1, we monitored the distance between the center of mass (CoM) of the heparin and the CoM of the binding site of the protein. In the first simulation (1st configuration), the residues considered to calculate the CoM are HIS8, LYS47, SER48, LYS50, THR187, TYR209 and ARG354. In the second simulation (2nd configuration), the residues considered to calculate the CoM are HIS8, ASN9, GLN10,

LYS11, ASN13, LYS15, LYS47, SER48, LYS50, GLN53, LEU135, THR187, TYR209, PHE326 and ARG354.

The number of residues in contact with the heparin molecule has been also calculated. A contact is considered if at least one atom of a specific residue is within 3 Å of any atom the heparin molecule.

The number of hydrogen bonds between the protein and the heparin fragment is also calculated. A hydrogen bond is considered if the donor-acceptor distance is within 3 Å and a acceptor-donor-hydrogen angle cutoff of 20°.

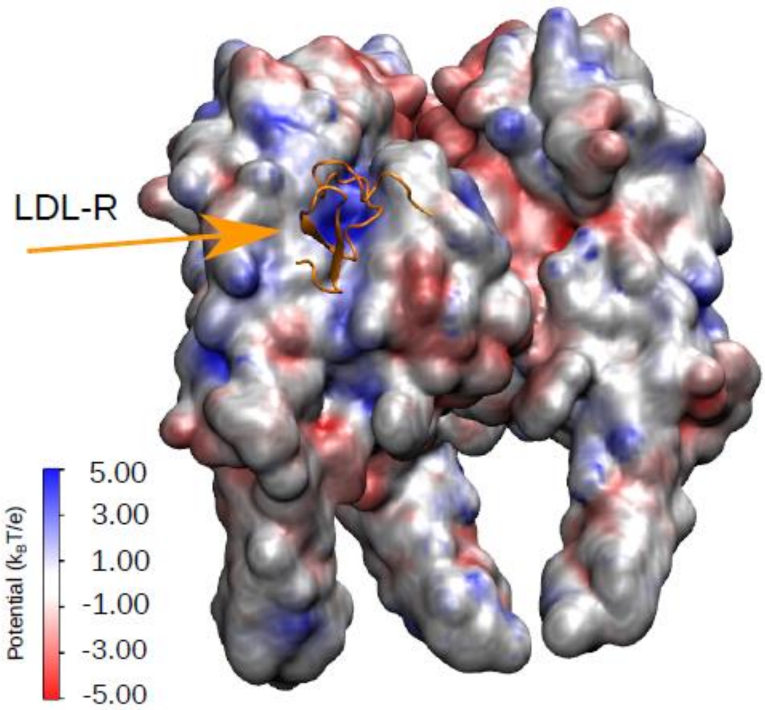

Figure S10. Binding site of the LDL-R receptor to the VSV-G protein. In this figure the position of the LDL-R receptor (in ribbon representation) has been superimposed to a complex with the VSV-G protein (extracted from the Protein Data Bank structure with PDB: ID 5OY9) and the electrostatic potential map of the protein.

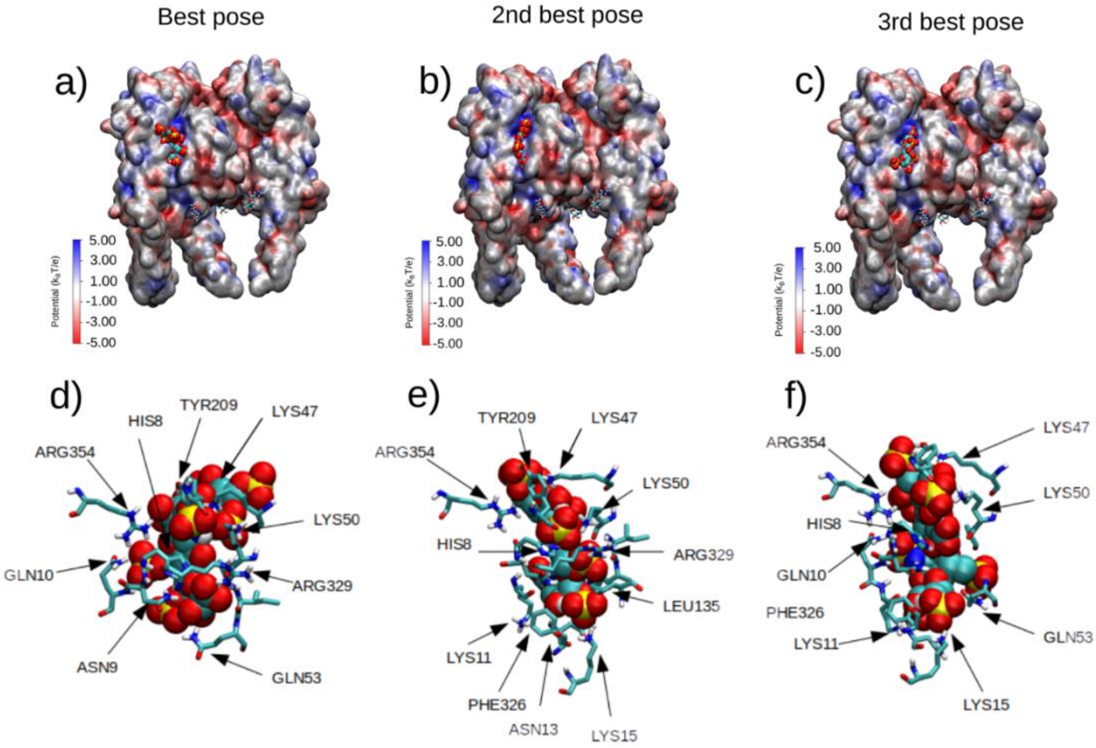

Figure S11. Results of docking calculations (three best poses). (a)-(c) Snapshots of the predicted protein-heparin complex (best pose, second, and third best poses respectively) with the electrostatic potential map superimposed over the protein structure. Heparin is shown in Van der Waals representation. (d)-(f) Detail of the protein residues in contact with heparin (residues within 3 Å of the heparin are shown). The protein residues are shown in Licorice representation. Color code: cyan (carbon), blue (nitrogen), red (oxygen), yellow (sulphur) and white (hydrogen).

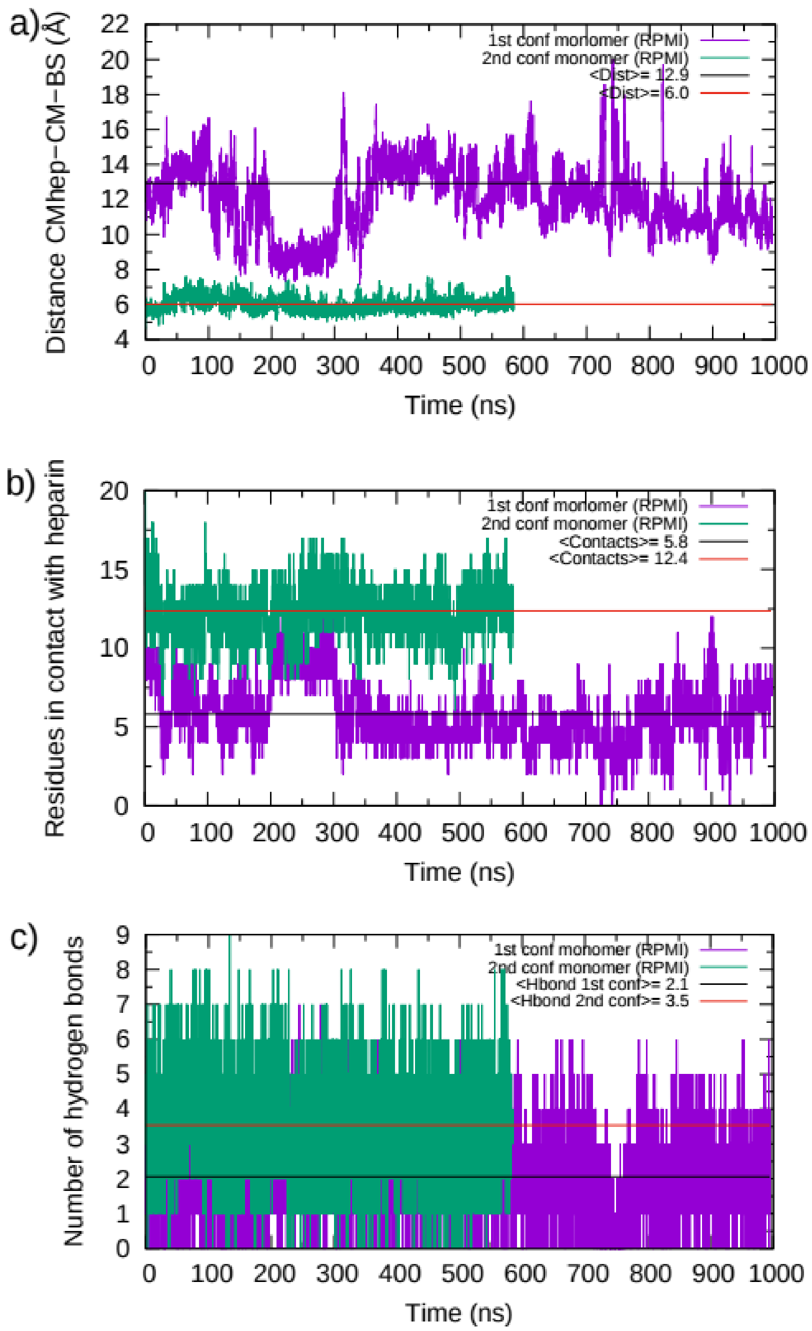

Figure S12. Results from MD simulations in Table S1. (a) Distance between the center of mass of the heparin and the center of mass of the binding site along the MD simulations. (b) Number of protein residues in contact with the heparin. (c) Number of heparin-protein intermolecular hydrogen bonds. The results indicated as “1st” and “2nd” correspond to MD simulations performed starting form the first- and second-best poses predicted by docking calculations. The equilibrium values (averaged form the last configurations) are also shown.

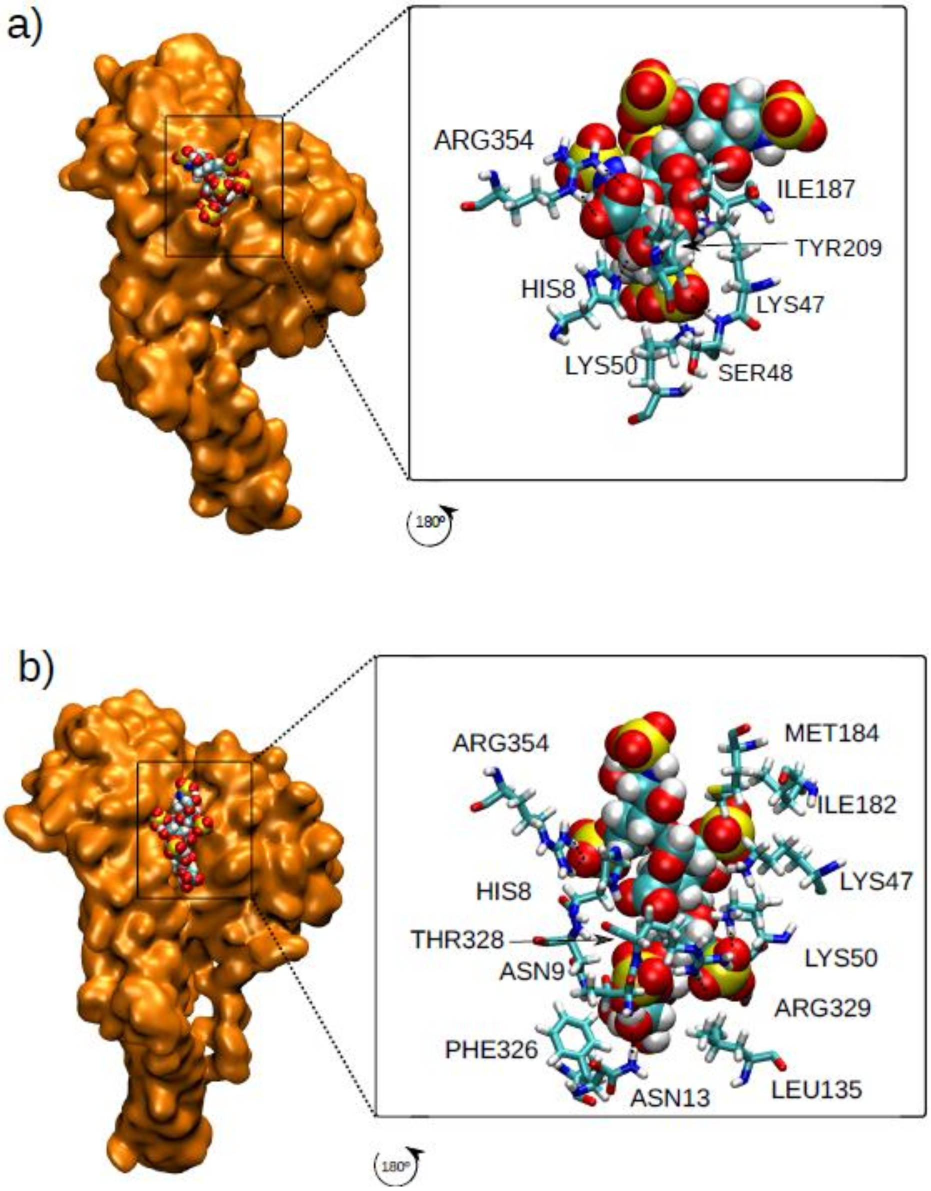

Figure S13. Snapshots from MD simulations in Table 1 (see also Figure S11) showing equilibrated configurations. The snapshot shown in (a) corresponds to the MD simulation starting form the best pose obtained in docking calculations, whereas the simulation shown in (b) corresponds to the MD simulation starting from the 2nd best pose from docking prediction. In these complexes, the protein monomer is shown in surface representation in orange color and the heparin is shown in Van der Waals representation. The zoom shows the detail of the protein residues in contact with heparin (residues within 3 Å of the heparin are shown). The protein residues are shown in Licorice representation. Color code: cyan (carbon), blue (nitrogen), red (oxygen), yellow (sulphur) and white (hydrogen).

Table S1. Characteristic parameters of the MD simulations of the VSV-G protein monomer and heparin fragment.

Initial condition	Num atoms	Box size (nm^3)	Number of ions					Simulation time (ns)
			Na^+	Cl^-	K^+	HCO_3^-	HPO_4^{-2}	
Best pose from docking	64007	11.1 X 7.9 X 7.1	63	42	2	9	2	995
2nd best pose from docking	79530	12.1 X 8.9 X 7.2	73	50	2	10	3	584

12. References

(1) Roederer, M. Interpretation of cellular proliferation data: Avoid the panglossian. *Cytom. A* **2011**, *79A*, 95-101.

(2) Jurrus, E.; Engel, D.; Star, K.; Monson, K.; Brandi, J.; Felberg, L. E.; Brookes, D. H.; Wilson, L.; Chen, J.; Liles, K.; Chun, M.; Li, P.; Gohara, D. W.; Dolinsky, T.; Konecny, R.; Koes, D. R.; Nielsen, J. E.; Head-Gordon, T.; Geng, W.; Krasny, R.; Wei, G.-W.; Holst, M. J.; McCammon, J. A.; Baker, N. A. Improvements to the APBS biomolecular solvation software suite. *Protein Sci.* **2018**, *27*, 112-128.

(3) Kozakov, D.; Hall, D. R.; Xia, B.; Porter, K. A.; Padhorny, D.; Yueh, C.; Beglov, D.; Vajda, S. The CLUSPRO web server for protein–protein docking. *Nat. Protoc.* **2017**, *12*, 255-278.
